# Fluid flow-induced left-right asymmetric decay of *Dand5* mRNA in the mouse embryo requires a Bicc1-Ccr4 RNA degradation complex

**DOI:** 10.1038/s41467-021-24295-2

**Published:** 2021-07-01

**Authors:** Katsura Minegishi, Benjamin Rothé, Kaoru R. Komatsu, Hiroki Ono, Yayoi Ikawa, Hiromi Nishimura, Takanobu A. Katoh, Eriko Kajikawa, Xiaorei Sai, Emi Miyashita, Katsuyoshi Takaoka, Kana Bando, Hiroshi Kiyonari, Tadashi Yamamoto, Hirohide Saito, Daniel B. Constam, Hiroshi Hamada

**Affiliations:** 1 Laboratory for Organismal Patterning, RIKEN Center for Biosystems Dynamics Research, Kobe, Hyogo Japan; 2 Ecole Polytechnique Fédérale de Lausanne (EPFL), School of Life Sciences, Lausanne, Switzerland; 3 Department of Life Science Frontiers, Center for iPS Cell Research and Application (CiRA), Kyoto University, Kyoto, Japan; 4 Laboratory for Animal Resources and Genetic Engineering, RIKEN Center for Biosystems Dynamics Research, Kobe, Hyogo Japan; 5 Laboratory for Immunogenetics, Center for Integrative Medical Sciences, Suehiro-cho, Yokohama Japan; 6 Cell Signal Unit, Okinawa Institute of Science and Technology, Kunigami-gun, Okinawa Japan

**Keywords:** Calcium signalling, Embryology, Body patterning, RNA decay

## Abstract

Molecular left-right (L-R) asymmetry is established at the node of the mouse embryo as a result of the sensing of a leftward fluid flow by immotile cilia of perinodal crown cells and the consequent degradation of *Dand5* mRNA on the left side. We here examined how the fluid flow induces *Dand5* mRNA decay. We found that the first 200 nucleotides in the 3′ untranslated region (3′-UTR) of *Dand5* mRNA are necessary and sufficient for the left-sided decay and to mediate the response of a 3′-UTR reporter transgene to Ca^2+^, the cation channel Pkd2, the RNA-binding protein Bicc1 and their regulation by the flow direction. We show that Bicc1 preferentially recognizes GACR and YGAC sequences, which can explain the specific binding to a conserved GACGUGAC motif located in the proximal *Dand5* 3′-UTR. The Cnot3 component of the Ccr4-Not deadenylase complex interacts with Bicc1 and is also required for *Dand5* mRNA decay at the node. These results suggest that Ca^2+^ currents induced by leftward fluid flow stimulate Bicc1 and Ccr4-Not to mediate *Dand5* mRNA degradation specifically on the left side of the node.

## Abstract (web summary)

Questioning what regulates left-right asymmetry breaking in the mouse node: the authors identify a 200 bp stretch of the Dand5 3’UTR where Bicc1 binds, and Cnot proteins downstream of calcium flow regulate the post-transcriptional regulation of Dand5 by Bicc1.

## Introduction

The first step in the establishment of left-right (L-R) asymmetry in vertebrate embryos is known as the L-R symmetry breaking event. In fish, frog, and mouse embryos, the breaking of L-R symmetry requires motile cilia that generate a unidirectional fluid flow at the L-R organizer, which corresponds to Kupffer’s vesicle in zebrafish, the gastrocoel roof plate in *Xenopus*, and the ventral node in mouse^1,2^. Whereas this fluid flow, known as nodal flow in the mouse, is essential for L-R symmetry breaking, its mechanism of action remains unknown. It may transport an unknown chemical determinant or it may generate a mechanical stimulus, either of which would then be sensed by the embryo.

Genetic studies have indicated that nodal flow is sensed by the transient receptor potential (TRP) cation channel Pkd2^3^ and its partner protein Pkd1l1^4^ present in immotile cilia of crown cells at the periphery of the node^5^. Flow sensation by Pkd2 is thought to result in the entry of Ca^2+^ ^6^ and the consequent degradation through an unknown mechanism of the mRNA for Dand5 (also known as Cerl2 or Cer2) in crown cells on the left side of the node^1,7^. Given that Dand5 is an antagonist of the extracellular signaling molecule Nodal, which is required for correct L-R patterning^8^, suppression of *Dand5* mRNA on the left side results in increased Nodal activity in crown cells on this side^9^. Flow-induced downregulation of *Dand5* mRNA specifically on the left side is the earliest molecular L-R asymmetry that has been detected at the node, and it, in turn, induces asymmetric expression of *Nodal* in the lateral plate^9^.

The specific factors responsible for the suppression of *Dand5* mRNA have remained unidentified. The only RNA binding protein known to be required for correct L-R patterning is Bicc1^10^. Bicc1 binds specific mRNAs via a tandem repeat of three ribonucleoprotein K homology (KH) domains and thereby inhibits their translation in a manner that is facilitated by self-polymerization of a sterile α motif (SAM) at the COOH-terminus of the protein^11,12^. Studies in *Drosophila* have shown that Bicc1 also interacts with the Ccr4-Not complex—the major cytoplasmic deadenylase responsible for mRNA turnover in eukaryotes^13,14^—through direct binding to its Not3/5 subunit^15^. In mammalian cells, lack of the Not3/5 ortholog Cnot3 is associated with increased mRNA stability^16,17^. However, a function for Bicc1 or the Ccr4-Not deadenylase complex in flow sensing, or in the regulation of mRNA translation or decay, during L-R patterning has not been described.

We have now identified an element in the 3′ untranslated region (3′-UTR) of *Dand5* mRNA that is responsible for the asymmetric decay of this mRNA at the node of the mouse embryo in a manner dependent on Bicc1 and the Ccr4-Not subunit Cnot3.

## Results

### A 3′-UTR reporter recapitulates the asymmetric distribution of *Dand5* mRNA at the node

**Fig. 1:**
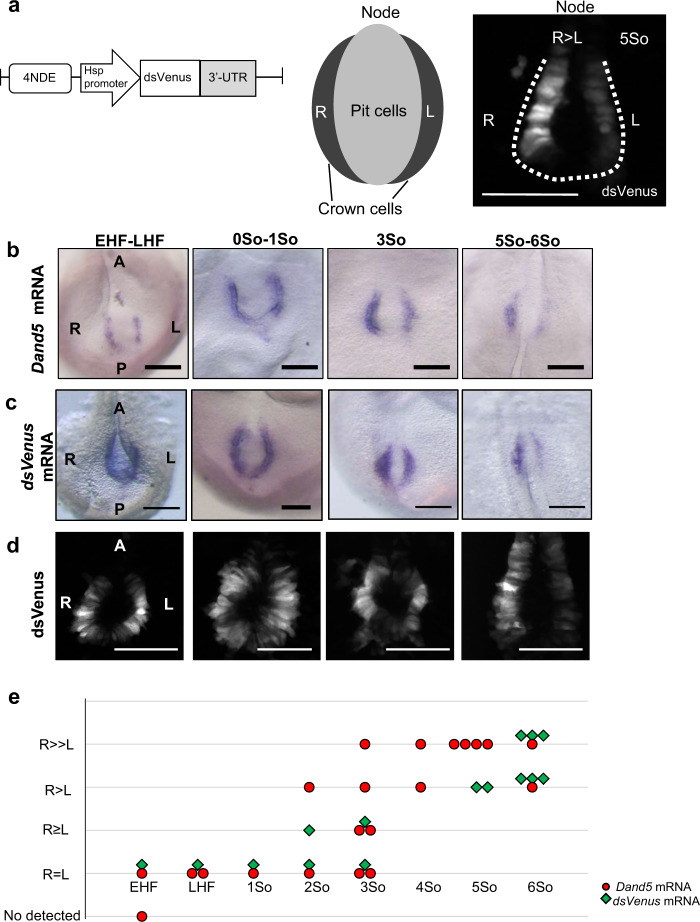
A 3′-UTR reporter recapitulates the asymmetric distribution of Dand5 mRNA at the node. **a** Schematic representation of the *NDE-Hsp-dsVenus-3*′*-UTR* transgene (left) and of the relative localizations of pit cells and crown cells at the node of mouse embryos (middle). The transgene contains the ORF for dsVenus linked to the DNA sequence for the 3′-UTR (1.2 kb) of mouse *Dand5* mRNA, and its expression is driven by the mouse *Hsp68* promoter and four copies of the crown cell–specific enhancer (NDE) of mouse *Nodal*. A typical pattern of dsVenus fluorescence detected at the node of a transgenic embryo at the five-somite (5So) stage is also shown (right), with the dashed line indicating the node region. Note that the fluorescence in crown cells is highly L-R asymmetric (R > L). Scale bars, 100 µm. **b** Whole-mount in situ hybridization (WISH) analysis of *Dand5* mRNA at the node of wild-type mouse embryos at the indicated developmental stages. Note that *Dand5* mRNA in crown cells is bilaterally equal at the early headfold (EHF) to late headfold (LHF) stages as well as the zero- to one-somite stages, but is asymmetric (R > L) at the three-somite and five- to six-somite stages. A and P, anterior and posterior, respectively. Scale bars, 100 µm. **c** WISH analysis of *dsVenus* mRNA in *NDE-Hsp-dsVenus-3*′*-UTR* transgenic embryos at the indicated stages. Scale bars, 100 µm. **d** Fluorescence images of dsVenus at the node of transgenic embryos at the indicated stages. Scale bars, 100 µm. Images shown in (**b**–**d**) are representative of at least two embryos at each stage. **e** Comparison of L-R asymmetry of *Dand5* mRNA in wild-type embryos and *dsVenus* mRNA in transgenic embryos at various developmental stages. Each point corresponds to one embryo. Note that the pattern of *dsVenus* mRNA recapitulates that of *Dand5* mRNA.

The L-R pattern of *Dand5* mRNA changes dynamically at the node of mouse embryos. At the early headfold stage, *Dand5* mRNA is induced specifically in crown cells on both sides of the node, and its distribution remains symmetric until the zero-somite stage (see Fig. 1). At the three-somite stage, the amount of *Dand5* mRNA has begun to decrease on the left side, resulting in a L-R asymmetric (R > L) distribution that is maintained at least until the six-somite stage. This decline in the abundance of *Dand5* mRNA on the left side coincides with a gradual increase in nodal flow^9,18^. Such observations have thus suggested that *Dand5* mRNA is degraded in crown cells on the left side of the node between the zero- and five-somite stages.

We first tested if the 3′-UTR of *Dand5* mRNA is able to recapitulate the distribution pattern of the mRNA at the node with the use of a transgene, *NDE-Hsp-dsVenus-3*′*-UTR*, that contains the open reading frame (ORF) for destabilized Venus (dsVenus) linked to the 1.2-kb DNA sequence corresponding to the 3′-UTR of *Dand5* mRNA (Fig. 1a). Expression of the *dsVenus-*3′*-UTR* cassette is driven by the mouse *Hsp68* promoter and the crown cell-specific enhancer (NDE) of mouse *Nodal*. As expected, *NDE-Hsp-dsVenus-3*′*-UTR* transgenic embryos expressed dsVenus specifically in node crown cells. Both *dsVenus* mRNA (Fig. 1c) and dsVenus fluorescence (Fig. 1a and d) were bilaterally equal at the early headfold and zero-somite stages but were asymmetric (R > L) at the three- and five-somite stages, recapitulating the pattern of *Dand5* mRNA (Fig. 1b and e). Hereafter, asymmetry of dsVenus fluorescence was examined at the five- to six-somite stages when the L-R asymmetry becomes obvious (Fig. 1e) unless indicated otherwise.

### The 3′-UTR of *Dand5* mRNA responds to the direction of fluid flow in a Pk d2-dependent manner

**Fig. 2:**
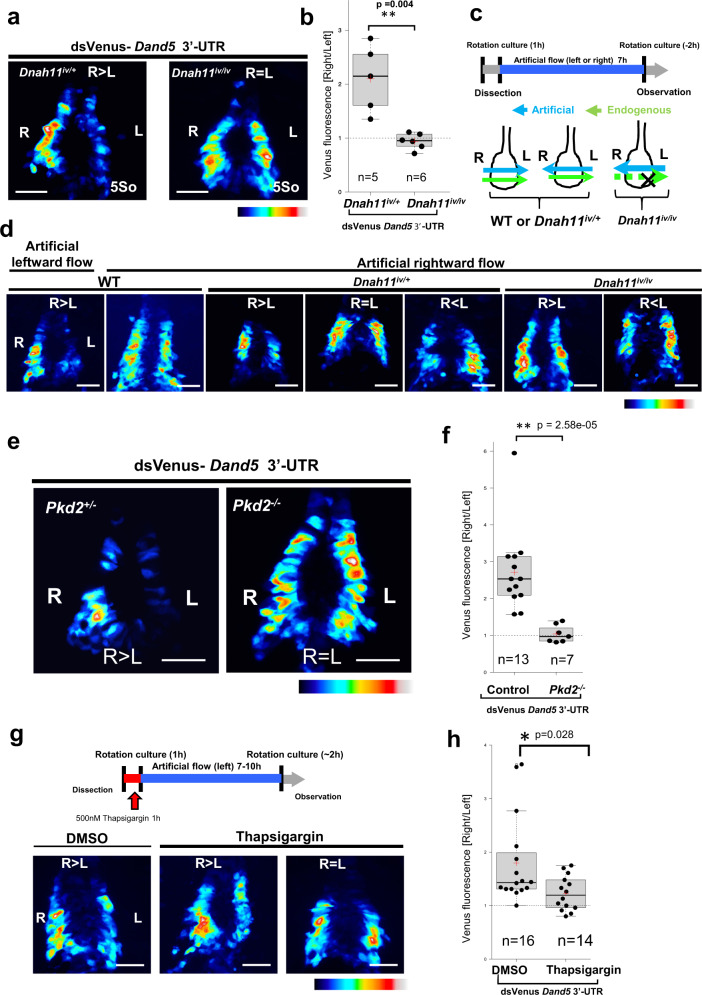
The 3′-UTR of *Dand5* mRNA responds to the direction of fluid flow in a Pkd2-dependent manner. **a** Fluorescence of dsVenus at the node of *Dnah11*^iv/+^ or *Dnah11*^iv/iv^ embryos harboring the *NDE-Hsp-dsVenus-3*′*-UTR* transgene at the five-somite stage. The color scale indicates the intensity of dsVenus fluorescence, with white corresponding to the maximal level. Images shown are representative of at least five embryos for each genotype. **b** Ratios of fluorescence on the right versus left side of the node at four- to six-somite stages shown for each genotype. The n values indicate the numbers of embryos analyzed. Red crosses indicate average values. **c** Design of artificial flow experiments. *Dnah11*^*+/+*^ (WT), *Dnah11*^iv/+^, or *Dnah11*^iv/iv^ embryos harboring the *NDE-Hsp-dsVenus-3*′*-UTR* transgene were cultured under the influence of a rightward or leftward artificial flow from the late headfold to five-somite stages. **d** Fluorescence of dsVenus at the node of representative embryos of the indicated genotypes after exposure to the leftward or rightward artificial flow as in (**c**). **e**, **f** Fluorescence of dsVenus at the node of *NDE-Hsp-dsVenus-3*′*-UTR* transgenic *Pkd2*^+/−^ or *Pkd2*^−/−^ embryos at the four- to six-somite stage (**e**) and its R/L ratios (**f**) quantified as in (**b**). **g** DsVenus fluorescence at the node of *NDE-Hsp-dsVenus-3*′*-UTR* embryos that were incubated at the four- to six-somite stage with thapsigargin (500 nM) or dimethyl sulfoxide (DMSO, vehicle), followed by administration of a leftward artificial flow. Scale bars, 50 µm. **h** Right/Left ratios of dsVenus fluorescence in groups of DMSO-treated versus thapsigargin-treated embryos from the experiment in (**g**) plotted as in (**b**) and (**f**). All scale bars, 50 µm. For the boxplots in (**b**, **f**, and **h**), the top and bottom lines of each box represent the 75th and 25th percentiles of the samples, respectively. The line inside each box represents the median of the samples. The upper and lower lines above and below the boxes are the whiskers. Statistical significance (*p*) was determined using the two-sided Wilcoxon rank sum test. Source data are provided as a Source Data file.

To test whether the *NDE-Hsp-dsVenus-3*′*-UTR* transgene is regulated by nodal flow, we introduced it into *iv/iv* (*Dnah11*^*iv/iv*^) mutant embryos, which lack the fluid flow^19^. In such embryos (*n* = 6/6), the transgene failed to give rise to an asymmetric (R > L) distribution of dsVenus fluorescence (Fig. 2a). Interestingly, the dsVenus pattern is bilaterally equal in all embryos examined (Fig. 2b and b), although L-R is often described as being randomized. This discrepancy may be due to the sensitivity of the assay employed. Mouse embryos in culture develop L-R asymmetry in response to the direction of an artificial fluid flow^20^. Exposure of wild-type (WT) embryos harboring the transgene to an artificial leftward fluid flow resulted in the development of R > L asymmetry of dsVenus fluorescence (*n* = 6/8) (Fig. 2c and d). By contrast, when exposed to an artificial flow toward the right side, such embryos instead developed bilaterally symmetric (R = L) dsVenus fluorescence (*n* = 6/6). Exposure of *iv/+* or *iv/iv* embryos to the artificial rightward flow resulted in the development of R < L (*n* = 3/6), R = L (*n* = 2/6), or R > L (*n* = 1/6) patterns of dsVenus fluorescence in the former embryos and in R < L (*n* = 2/7), R = L (*n* = 4/7), and R > L (*n* = 1/7) patterns in the latter (Fig. 2c and d, Supplementary Fig. 1a). These results collectively suggested that the 3′-UTR of *Dand5* mRNA responds to the direction of fluid flow.

*Pkd2* encodes an ion channel that allows the passage of cations including Ca^2+^ ^21^, and is required for L-R patterning in the mouse embryo^3^. In *Pkd2*^–/–^ embryos, the *NDE-Hsp-dsVenus-3*′*-UTR* transgene was expressed bilaterally at the node (*n* = 7/7) (Fig. 2e, f). Similarly, treatment of WT embryos harboring the transgene for 1 h with thapsigargin, which blocks Ca^2+^ release from the endoplasmic reticulum^22^, resulted in half of the tested embryos (*n* = 7/14) failing to downregulate the reporter mRNA on the left side in response to an artificial leftward flow, disrupting the L-R asymmetric pattern of dsVenus fluorescence (Fig. 2g and h). Longer treatment with thapsigargin resulted in developmental arrest. Together, these results implicate Ca^2+^ in the flow-induced decay of *Dand5* mRNA.

### A conserved 200-nucleotide region of the proximal 3′-UTR is required for L-R asymmetry of *Dand5* mRNA

**Fig. 3:**
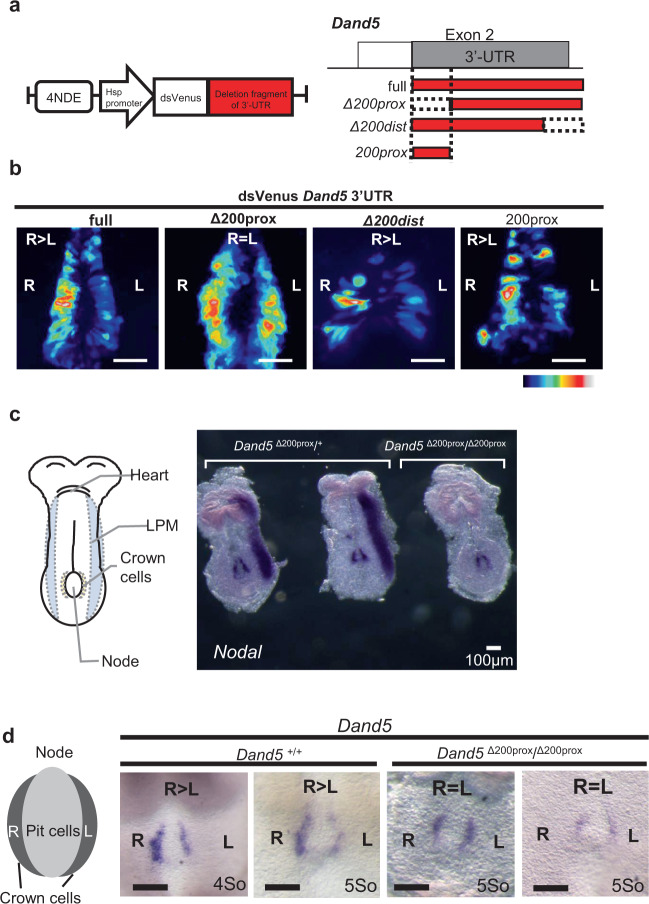
A 200-nucleotide conserved proximal region of the *3*′-UTR is required for L-R asymmetric accumulation of *Dand5* mRNA. **a** Schematic representation of the *NDE-Hsp-dsVenus* transgene fused to the *Dand5 3*′-UTR *(left)* or to a series of various *3*′*-UTR* fragments (*right*). **b** Fluorescence of dsVenus at the node of four- to six-somite stage mouse embryos harboring the indicated transgene (see also Supplementary Fig. 1b). Images are representatives of at least three embryos for each transgene. Scale bar, 50 µm. **c** WISH analysis of *Nodal* expression in *Dand5*^Δ200prox/+^ and *Dand5*^Δ200prox/Δ200prox^ embryos at embryonic day (E) 8.0. Left-sided expression of *Nodal* in LPM was lost in the *Dand5*^Δ200prox/Δ200prox^ embryo. Scale bar, 100 µm. Images are representatives of at least four embryos for each genotype. **d** WISH analysis of *Dand5* expression at the node of *Dand5*^+/+^ and *Dand5*^Δ200prox/Δ200prox^ embryos at four- to five-somite stages (E8.0). Two representative embryos are shown for each genotype. L-R asymmetry of *Dand5* mRNA at the node was lost in the *Dand5*^Δ200prox/Δ200prox^ embryo. Scale bars, 100 µm.

To map the sequence within the 3′-UTR required for generation of L-R asymmetry of *Dand5* mRNA, we first compared the DNA sequences corresponding to the 3′-UTR (~1.2 kb) among mammalian *Dand5* genes. We found that the 5′-most 200 nucleotide region (200prox) was substantially conserved (Supplementary Fig. 2). To test whether this region is required for L-R asymmetric gene expression, we first deleted it from the *NDE-Hsp-dsVenus-3*′*-UTR* transgene (Fig. 3a). The resulting *NDE-Hsp-dsVenus-3*′*-UTR-Δ200prox* transgene yielded bilaterally symmetric dsVenus fluorescence at the node (Fig. 3b, and Supplementary Fig. 1b) (*n* = 5/6). On the other hand, deletion of the 3′-most 200 nucleotides from the 3′-UTR (*Δ200dist* in Fig. 3a) did not perturb asymmetric dsVenus fluorescence (Fig. 3b) (*n* = 3/3). Furthermore, the 5′-most 200 nucleotide region (*200prox* in Fig. 3a) alone gave rise to asymmetric dsVenus fluorescence (*n* = 4/5) (Fig. 3b). Prompted by these results, we applied CRISPR-based editing to delete this proximal 200-nucleotide region of the *Dand5 3*′-UTR in the mouse genome, thereby generating the *Dand5*^Δ200prox^ allele (Supplementary Fig. 3). WISH analysis revealed that whereas bilateral *Nodal* expression at the node was maintained, left-sided *Nodal* expression in lateral plate mesoderm (LPM) was lost in *Dand5*^Δ200prox/Δ200prox^ embryos (*n* = 4/4)(Fig. 3c), as expected if *Dand5* mRNA remains stable on both sides of the node. Among eight *Dand5*^Δ200prox/+^ embryos examined, one embryo lost *Nodal* expression in LPM, suggesting that the *Dand5*^Δ200prox^ allele may act dominantly in rare instances. WISH analysis also showed that *Dand5* mRNA was not distributed asymmetrically in *Dand5*^Δ200prox/Δ200prox^ embryos (*n* = 5/5) (Fig. 3d). Together, these results indicated that the 200-nucleotide proximal region of the 3′-UTR is involved in the L-R asymmetric pattern of *Dand5* mRNA in node crown cells.

### Flow-induced decay of *Dand5* mRNA depends on Bicc1

**Fig. 4:**
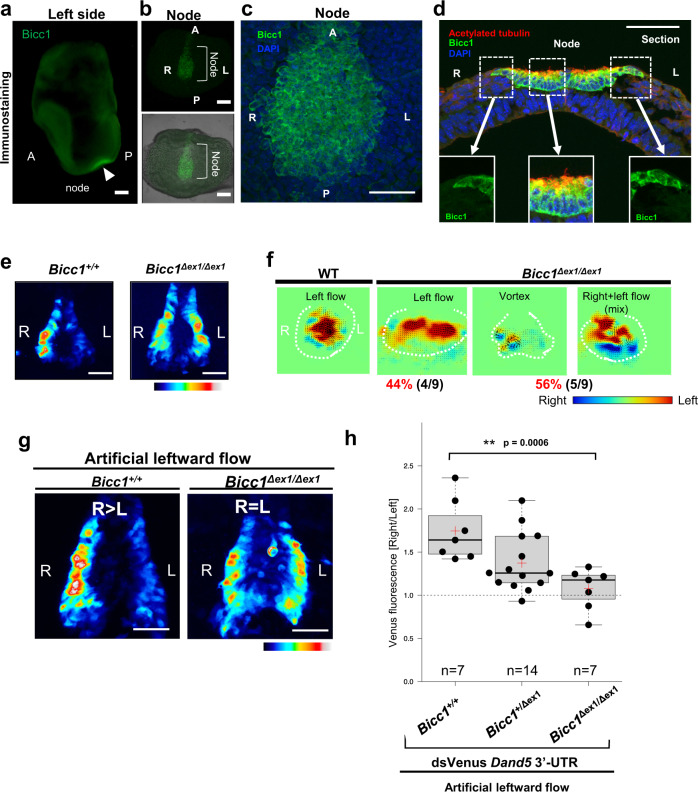
Flow-induced decay of *Dand5* mRNA depends on Bicc1. **a**-**d** Immunofluorescence staining of Bicc1 in node cells of a WT embryo at E8.0 (**a**), or its node region at higher magnification (**b**, **c**), and in a transverse section of the node (**d**). Nuclei were stained with 4’,6-diamidino-2-phenylindole (DAPI) in (**c**) and (**d**), and acetylated tubulin was also immunostained in (**d**) to reveal cilia. Scale bars in (**a**, **b**) and (**c**, **d**) are 100 µm and 50 µm, respectively. Images shown in (**a**-**d**) are representative of at least two independent experiments. **e** dsVenus fluorescence at the node of four- to six-somite stage WT and *Bicc1*^Δex1/Δex1^ embryos harboring the *NDE-Hsp-dsVenus-3*′*-UTR* transgene. Scale bars, 50 µm. Images shown are representative of six embryos for each genotype. **f** Nodal flow in WT and *Bicc1*^Δex1/Δex1^ mouse embryos at three- to five-somite stages as revealed by particle image velocimetry (PIV) analysis. The dashed white line indicates the outline of the node, small arrows in the node region indicate the direction and velocity of flow at those positions. The relative color scale indicates the magnitude of flow velocity (leftward in red, rightward in blue). Note that leftward laminar flow was lost in five out of nine *Bicc1*^Δex1/Δex1^ embryos, whereas the four others showed normal leftward flow. **g** *Bicc1*^*+/+*^ and *Bicc1*^Δex1/Δex1^ embryos harboring the *NDE-Hsp-dsVenus-*3′*-UTR* transgene were cultured in the presence of an artificial leftward flow as in Fig. 2c, after which the L-R pattern of dsVenus fluorescence at the node was examined. Scale bars, 50 µm. **h** Quantification of Right/Left ratios of dsVenus fluorescence at the node of *NDE-Hsp-dsVenus-3*′*-UTR* embryos of the indicated genotypes after culture in leftward artificial flow as in (**g**). The n values indicate the numbers of embryos analyzed, and red crosses indicate average values. For the boxplots, the top and bottom lines of each box represent the 75th and 25th percentiles of the samples, respectively. The line inside each box represents the median of the samples. The upper and lower lines above and below the boxes are the whiskers. Statistical significance (p) was determined using the two-sided Wilcoxon rank sum test. Source data are provided as a Source Data file. Note that the artificial leftward fluid flow failed to rescue the impaired L-R pattern of dsVenus fluorescence in the *Bicc1*^Δex1/Δex1^ embryos.

We hypothesized that a specific factor (or factors) in crown cells binds to the proximal portion of the 3′-UTR to mediate the decay of *Dand5* mRNA preferentially on the left side of the node. Among candidates for such a factor, we focused on Bicc1, an RNA binding protein with KH domains for RNA binding and a SAM domain for polymerization^12,23^, given that it is specifically expressed at the node of mouse embryos and that *Bicc1*^–/–^ embryos manifest laterality defects^10^. Whole-mount immunofluorescence analysis revealed that pit cells and crown cells at the node express Bicc1 in an apparently L-R symmetric manner (Fig. 4a–c). Moreover, whereas *Dand5* mRNA is localized predominantly at the apical side of crown cells^24^, Bicc1 was detected both apically and basally (Fig. 4d). To evaluate potential genetic interaction between *Bicc1* and *Dand5*, we deleted the first exon of *Bicc1* using the CRISPR/Cas9 system (Supplementary Fig. 4a). The fluorescence of dsVenus in the resulting *Bicc1*^Δex1/Δex1^ embryos harboring the *NDE-Hsp-dsVenus-3*′*-UTR* transgene was bilaterally symmetric (n = 6/6) (Fig. 4e, Supplementary Fig. 1c), suggesting that the decay of *Dand5* mRNA on the left side is impaired in the absence of Bicc1. Similar to our previous observations with *Bicc1*^–/–^ embryos^10^, the basal body of motile cilia at the node failed to shift posteriorly in half of the *Bicc1*^Δex1/Δex1^ embryos examined (*n* = 3/6) (Supplementary Fig. 4b and c). Consistent with this finding, the leftward flow at the node was disrupted in half of *Bicc1*^Δex1/Δex1^ embryos (*n* = 5/9), with the other half (*n* = 4/9 embryos) showing normal leftward flow (Fig. 4f). Nonetheless, an artificial leftward fluid flow failed to rescue the pattern of dsVenus fluorescence in *Bicc1*^Δex1/Δex1^ embryos examined (*n* = 7/7), suggesting that the mutant embryos are unable to respond to the fluid flow (Fig. 4g and h).

### Bicc1 binds to the 3′-UTR of *Dand5* mRNA

**Fig. 5:**
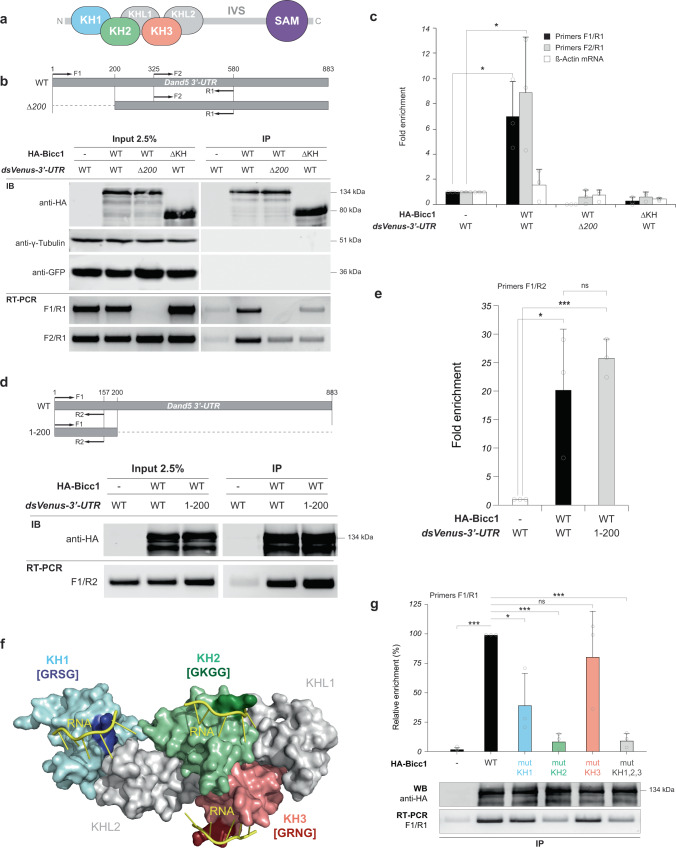
Bicc1 binds to the proximal 3′-UTR of *Dand5* mRNA via its KH1 and KH2 domains. **a** Domain organization of Bicc1. KH: K homology; KHL: KH-like; IVS: Intervening sequence; SAM: Sterile α motif. **b** Co-immunoprecipitation of mRNAs with HA-Bicc1 in HEK293T cells, and PCR primers used to detect *Dand5* reporter mRNAs by RT-PCR or -qPCR analysis. The primers F1/R1 detect only *dsVenus-3*′*-UTR* mRNA, whereas the F2/R1 primers also detect *dsVenus-3*′*-UTRΔ200prox*. Extracts of cells co-expressing the indicated reporter constructs and HA-Bicc1 (WT) or HA-Bicc1ΔKH were subjected to anti-HA immunoprecipitation. Immunoprecipitates (IP) and portions of the input extracts were subjected to anti-HA Western blot (WB) and to RT-PCR analysis. **c** RT-qPCR analysis of immunoprecipitates as in (**b**), but including β-actin mRNA (negative control). Data represent the ratio of co-immunoprecipitated mRNA to the input relative to the corresponding condition without HA-Bicc1. Source data are provided as a Source Data file. **d** Co-immunoprecipitation of reporter mRNAs with HA-Bicc1 in HEK293T cells. Extracts of cells expressing the indicated constructs were prepared as in (**b**). **e** RT-qPCR analysis of immunoprecipitates from the experiments in (**d**). Results are expressed as the ratio of co-immunoprecipitated mRNA to the input, normalized to the corresponding condition without HA-Bicc1. Source data are provided as a Source Data file. **f** Three-dimensional model of the mouse Bicc1 KH domain repeat in surface representation. Putative RNA-binding GXXG motifs (where at least one X is a basic residue) are highlighted. **g** RT-qPCR and Western blot analyses of immunoprecipitates prepared as in (**c**) from HEK293T cells expressing the *dsVenus-*3′*-UTR* construct and wild-type HA-Bicc1 (WT) or mutant versions in which the GXXG motif of individual KH domains (mutKH1, mutKH2, or mutKH3) or all three KH domains (mutKH1,2,3) were replaced with GDDG. RT-qPCR data represent the ratio of co-immunoprecipitated mRNA to the input and are expressed as a percentage of the value for HA-Bicc1(WT). Data in (**c**), (**e**) and (**g**) are means + SD from three independent experiments. *p < 0.05, **p < 0.01, ***p < 0.001 (two-sided Student’s *t*-test). Source data are provided as a Source Data file.

To assess whether the 200-nucleotide proximal portion of the *3*′-UTR of *Dand5* mRNA mediates the binding of Bicc1, we ligated *dsVenus-3*′*-UTR*, *dsVenus-3*′*-UTRΔ200prox* or *dsVenus-3*′*-UTR(200prox)* mRNA to a ubiquitous promoter for co-expression with hemagglutinin epitope (HA)-tagged mouse Bicc1 in HEK293T cells. Reverse transcription (RT) and quantitative polymerase chain reaction (qPCR) analysis of anti-HA immunoprecipitates and input extracts revealed that the *dsVenus-3*′*-UTR* and *dsVenus-3*′*-UTR(200prox)* mRNAs were enriched in the HA-Bicc1 precipitates (Fig. 5a–e). By contrast, a truncated form of Bicc1 lacking all KH domains (ΔKH)^10^ failed to recruit the WT *dsVenus-3*′*-UTR* mRNA (Fig. 5b and c). In KH domains, a conserved GXXG motif (where at least one X is a basic residue) binds single-stranded RNA sequences of 4 nucleotides and orients them toward an adjacent groove for nucleobase recognition^11,23^ (Fig. 5f). To evaluate the contribution of individual GXXG motifs to RNA binding, we mutated them to GDDG^25^. Mutation of the KH1 domain reduced the binding of Bicc1 to the *dsVenus-3*′*-UTR* reporter mRNA more than twofold, and mutation of the KH2 domain or of all three KH domains together nearly abolished it. By contrast, mutation of the KH3 domain alone had no significant effect on binding (Fig. 5g). Together, these results suggest that binding of Bicc1 to the 3′-UTR of *Dand5* mRNA is mediated by the 200-nucleotide proximal portion of the 3′-UTR and the KH1 and KH2 domains of Bicc1. However, binding to HA-Bicc1 alone did not reduce *dsVenus-3*′*-UTR* reporter mRNA translation (Fig. 5b, anti-GFP blot), indicating that the regulation of Bicc1 activity by nodal flow in vivo requires additional factors that are absent or inactive in HEK293T cells.

### Bicc1 specifically recognizes a conserved 8-mer motif in the *Dand5 3*′*-UTR*

**Fig. 6:**
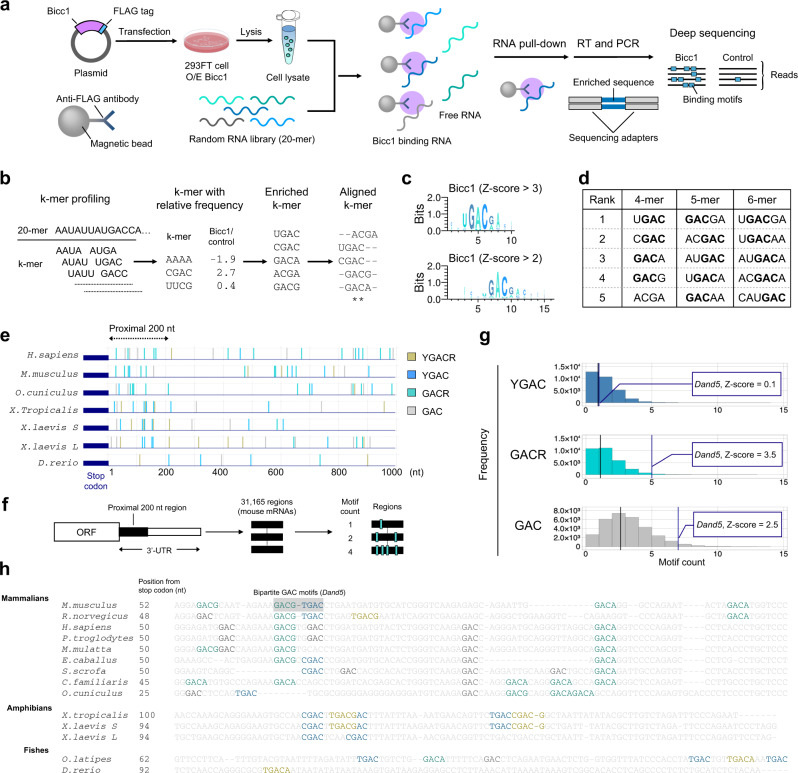
Screen for Bicc1-binding motifs in RNA. **a** Schematic representation of RNA Bind-n-Seq (RBNS), which determines RNA motifs enriched by target proteins with the use of a random RNA sequence library. 293FT cells were transfected with a plasmid for overexpression (O/E) of FLAG-tagged Bicc1. Cell lysates containing the Bicc1-FLAG protein were then mixed with a random RNA sequence library, and resulting RNA-protein complexes were isolated by immunoprecipitation with magnetic bead–conjugated antibodies to FLAG. Finally, the isolated RNA sequences were converted to a DNA library by RT-PCR for deep sequencing. **b** Analysis of the RBNS data set. The number of each k-mer (where *k* = 4, 5, or 6) RNA sequence was compared between cells transfected with the Bicc1-FLAG expression plasmid and those subjected to mock transfection (control). **c** Motif logos generated from aligned hexamers that were enriched by Bicc1-FLAG. **d** 4-mer, 5-mer, and 6-mer sequences ranked by their relative frequencies in Bicc1-FLAG versus control RBNS data. **e** Maps of GAC-containing motifs in the 3′-UTR of *Dand5* mRNAs for the indicated species. **f** Schematic representation of metagene analysis for the 200-nucleotide proximal region of the 3′-UTR of mouse mRNAs. A total of 31,165 regions extracted from mouse genes (mm10) was searched with the indicated target motifs. **g** Histogram of motif frequency revealed by metagene analysis. The vertical lines indicate the averaged frequency of each target motif (black) and the frequency of each target motif in the 200-nucleotide proximal region of the 3′-UTR of Dand5 mRNA (blue), respectively. **h** Multiple sequence alignment of a conserved segment within the proximal 200 nucleotides of mammalian, amphibian, and fish *Dand5* 3′*-UTRs*. Colors highlight GAC motifs.

To identify where Bicc1 binds the 3′-UTR of *Dand5* mRNA, we performed RNA Bind-n-Seq (RBNS) analysis^26^ with a random 20-mer RNA library using lysates of 293FT cells expressing FLAG–tagged Bicc1 (Fig. 6a). Analysis of Bicc1-interacting RNA 20-mers showed that the GAC-containing motifs were highly enriched (Fig. 6b and c), with YGAC (where Y represents a pyrimidine) and GACR (where R represents a purine) motifs showing higher enrichment scores than other GAC motifs (Fig. 6d). Both in mammalian and *Xenopus Dand5 3*′-UTRs, GACR and YGAC motifs were most enriched in the 200-nucleotide proximal regions (Fig. 6e). Metagene analysis with the 200-nucleotide proximal region of the 3′-UTR of mouse mRNAs revealed that the frequency of GACR and GAC was significantly enriched in the *Dand5* 3′-UTR (Fig. 6f and g). Furthermore, among 8-mer sequences, GACN_1-2_GAC sequences containing two GAC motifs were also enriched (Supplementary Fig. 5), suggesting a preference of Bicc1 for GACR and YGAC motifs alone or in combination. The juxtaposed GACR and YGAC motifs appear to be conserved in mammals, and a similar bipartite motif(s) is found in *Xenopus* and medaka fish. Thus, the *Xenopus* sequence CGACUUGAC at this position corresponds to two YGAC motifs, followed by the UGACCGACG in close proximity (Fig. 6h, Supplementary Fig. 6). The consensus motif GACN_1-3_GAC thus appeared as a likely candidate to mediate the recruitment of mammalian Bicc1 (Fig. 6h). Zebrafish *Dand5* mRNA and other target mRNAs of Bicc1 lack such a bipartite motif, although they contain GAC motifs in their 3′-UTRs (Supplementary Fig. 6). Bicc1 may recognize different panels of sequences by using distinct combinations of its three KH domains.

Secondary structure prediction based on sequence conservation revealed that the 3′ half of this GACGUGAC motif (UGAC in murine *Dand5 3*′-UTR) may be buried in a stable hairpin (Supplementary Fig. 7a). To assess Bicc1 binding, 45- to 225-nucleotide RNA probes comprising this motif were fluorescently labeled and incubated in vitro with increasing concentrations of recombinant GST-KH domains (Supplementary Fig. 7b). Analysis by Electrophoretic Mobility Shift Assays (EMSA) revealed that GST-KH shifted all three probes into several ribonucleoprotein (RNP) complexes (Supplementary Fig. 7c), with the highest affinity complex RNP_1_ showing an apparent Kd of 233 nM for the 225-nucleotide probe, compared to only 120 nM for the shorter 35-157 and 66-110 probes, respectively. Additional RNPs migrating with slower mobility only formed at elevated GST-KH concentrations with all three RNA probes, suggesting they likely represent higher-order complexes. To assess specificity, we performed competition experiments by incubating pre-assembled RNPs with increasing amounts of non-fluorescent competitor transcripts. The half maximal inhibitory concentration (IC50) of unlabeled *Dand5 3*′*-UTR*_*1-225*_ RNA was observed at 283 nM, whereas the distal *Dand5 3*′*-UTR*_*226-425*_ fragment, which is similar in size but contains no GAC motifs, was not competitive (IC50 > 1 µM) (Supplementary Fig. 7b and d). Similarly, unlabeled 35-157 and 66-110 fragments competed with fluorescent probes, showing IC50 values of 191 nM and 203 nM respectively, whereas control fragments of similar size from the distal 3′-UTR did not (Supplementary Fig. 7e and f). These results confirm that the proximal segment of the *Dand5 3*′*-UTR* has a higher affinity for Bicc1 than the distal region.

**Fig. 7:**
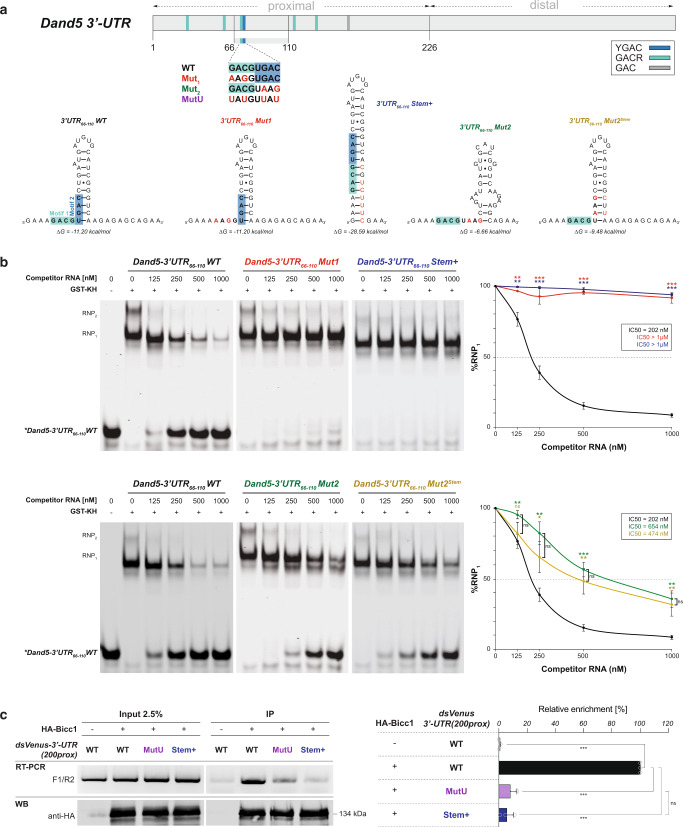
A conserved GACGUGAC motif recruits Bicc1 to the proximal *Dand5 3*′*-UTR*. **a** In vitro-transcribed *Dand5 3*′*-UTR*_*66-110*_ RNA and mutant versions used as competitors in EMSA assays to assess the specificity of Bicc1 binding. Their secondary structures and minimum free energies (ΔG) were predicted by the RNAfold server at http://rna.tbi.univie.ac.at/cgi-bin/RNAWebSuite/RNAfold.cgi. **b** EMSA analysis of competitive binding of Bicc1 KH domains to in vitro transcripts of WT or mutant *Dand5* 3′-UTR_66-110_ against the fluorescent wild-type *Dand5* 3′-UTR_66-110_ RNA probe. Increasing amounts of unlabeled competitors were added to pre-assembled complexes of fluorescent probe with recombinant GST-KH fusion protein. The % of residual RNP_1_ relative to the indicated competitor RNA concentration, and the half maximal inhibitory concentrations (IC50) of each competitor are indicated in the graphs to the right. *P* values (asterisks) above the curves refer to differences between each mutant versus the WT RNA, whereas differences between mutants (n.s.: not significant) are marked by brackets. **c** RT-PCR analysis of WT and mutant *dsVenus-Dand5* 3′*-UTR(200prox)* mRNAs in anti-HA immunoprecipitates of HA-Bicc1 from cytoplasmic extracts of HEK293T cells expressing the indicated constructs. Control anti-HA Western blots (WB) of inputs (2.5%) and immunoprecipitates (IP) are shown below, and RT-qPCR analysis of immunoprecipitates to the right. Enrichment of co-immunoprecipitated mRNA relative to the input is shown as the percentage of enrichment of WT mRNA (100%). All quantifications represent means ± SD from three independent experiments. *p < 0.05, **p < 0.01, ***p < 0.001 (two-sided Student’s *t*-test). Source data are provided as a Source Data file.

To evaluate the contribution of the GACGUGAC motif to Bicc1 binding, we tested the influence of specific mutations in the short *Dand5 3*′*-UTR*_*66-110*_ fragment (Fig. 7a). Analysis of the *3*′*-UTR*_*66-110*_*Mut1* RNA probe by competitive EMSA showed that mutation of GACG to AAGG abolished competitive GST-KH binding (IC50 > 1 µM) (Fig. 7b). Likewise, the *3*′*-UTR*_*66-110*_*Stem*+ probe that was engineered to mask the GACG motif in an elongated stem, lacked competitive binding activity (IC50 > 1 µM). Competitive binding also sharply decreased upon mutation of UGAC to UAAG, as shown by the *3*′*-UTR*_*66-110*_*Mut2* probe (IC50 = 654 nM). To rule out a non-specific inhibitory effect resulting from disruption of the stem, we generated *3*′*-UTR*_*66-110*_*Mut2*^*Stem*^ where a UAAG-complementary sequence should restore stem-loop formation (Fig. 7a). No difference was observed between these two mutants in their affinity for Bicc1, suggesting that specific Bicc1 binding depends on the GACGUGAC sequence and not just on the secondary structure (Fig. 7b, bottom panels). As another independent specificity control, we used the GACGUGAC octamer to replace a similar, though non-specific, GCCCUGGG sequence after two adenosines in the distal 226-270 fragment (Supplementary Fig. 8a). Interestingly, the resulting *3*′*-UTR*_*226-270*_*8-mer* RNA probe competed for Bicc1 KH domains with high affinity (IC50 = 392 nM), indicating that one GACGUGAC sequence is sufficient to confer specific binding (Supplementary Fig. 8b).

To directly test the role of the GACGUGAC motif in the *dsVenus-3*′*-UTR* reporter, we replaced critical nucleotides by uridines (UAUGUUAU). Interestingly, the resulting mutU reporter mRNA failed to bind Bicc1 above background levels (Fig. 7c). Similarly, the 3′UTR_86-110_Stem+ reporter which was designed to mask the GACGUGAC octamer in an elongated stem failed to coimmunoprecipitate with HA-Bicc1 (Fig. 7c), confirming the loss of binding observed in competitive EMSA assays (Fig. 7b). Together, these results indicate that the specific recruitment of Bicc1 to the *Dand5 3*′*-UTR* is mediated by the bipartite GACGUGAC motif. The juxtaposition of GACG and UGAC within this motif likely explains why its specific recognition by Bicc1 involves both the KH2 and the KH1 domains (Supplementary Fig. 9).

### Decay of *Dand5* mRNA on the left side of the node requires the Ccr4-not deadenylase complex

**Fig. 8:**
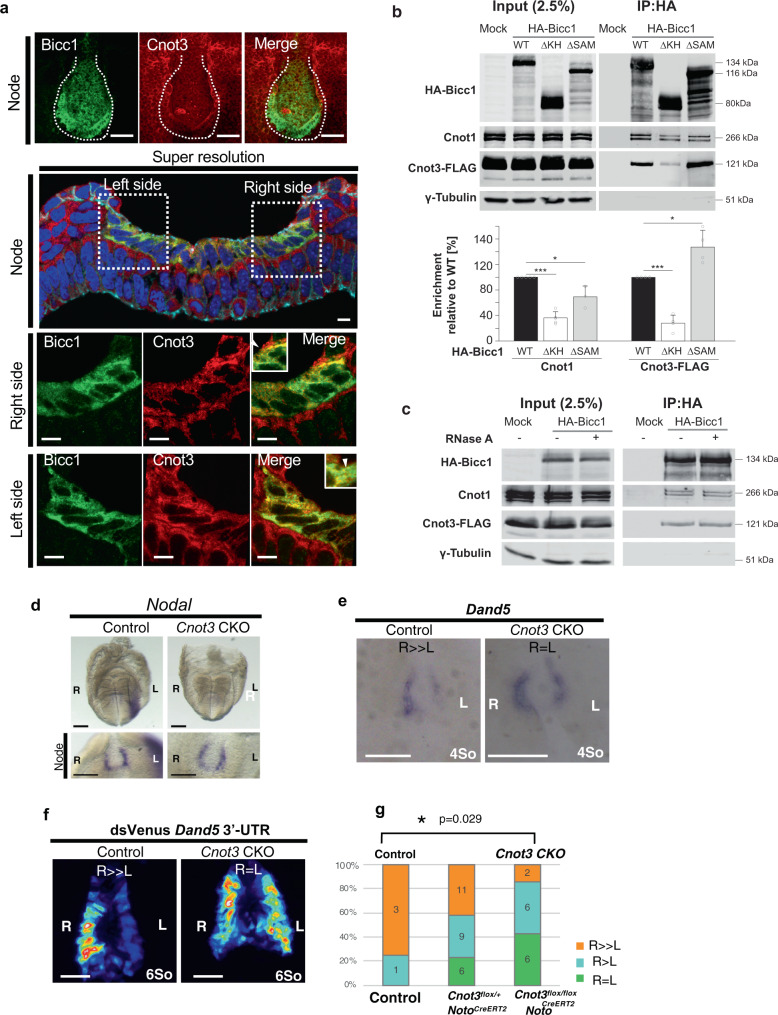
Decay of *Dand5* mRNA on the left side of the node requires the Ccr4-Not deadenylase complex. **a** Immunofluorescence staining of Bicc1 and Cnot3 in node cells of the WT mouse embryo at E8.0 shown as whole mounts (top row) and in a transverse section (second row). Nuclei were stained with 4’,6-diamidino-2-phenylindole (DAPI). Regions of the node indicated by the dotted rectangles are shown in bottom rows at higher magnification. Note that Bicc1 and Cnot3 proteins are co-localized in the cytoplasm (arrowheads). Scale bars in the top panels, 50 µm. Scale bars in the super resolution panels, 5 µm. Images shown are representative of at least two independent experiments. **b** Extracts of HEK293T cells transfected with expression plasmids for Cnot3-FLAG and either WT or the indicated mutant forms of HA-Bicc1 (or with the corresponding empty vector, Mock) were subjected to immunoprecipitation with antibodies to HA. The resulting immunoprecipitates as well as a portion (2.5%) of the original cell extracts were then subjected to immunoblot analysis with antibodies against HA, Cnot1, FLAG, or γ-tubulin (loading control). The enrichment of Cnot subunits relative to the indicated HA-Bicc1 proteins in the IP fractions is shown as a percentage of the value for HA-Bicc1 WT. Data are means ± SD from at least three independent experiments. The precise value of replicate numbers is indicated by grey circles. For the anti-Cnot1 Western blot, *n* = 5 for ΔKH, *n* = 3 for ΔSAM and n = 5 for wild-type. For anti-FLAG Western blots, *n* = 4 for ΔKH, ΔSAM and wild-type. *p < 0.05, **p < 0.01, ***p < 0.001 (two-sided Student’s *t*-test). Source data are provided as a Source Data file. **c** Co-immunoprecipitation of Cnot1 and Cnot3-FLAG with HA-Bicc1 before and after RNase A treatment of cell extracts was performed as in (**b**). **d** WISH analysis of Nodal expression in control and *Cnot3* CKO embryos at E8.0. The node region of the same embryo is shown below. Note that *Nodal* expression at the node was maintained at the node but that in LPM was lost in the mutant embryo. Scale bars, 100 µm. **e** WISH analysis of *Dand5* mRNA in control and *Cnot3* CKO embryos at four- to six-somite stages. The R > L asymmetry of *Dand5* mRNA in the control embryo is attenuated or lost in the *Cnot3* CKO embryos. Scale bars, 100 µm. **f** Fluorescence of dsVenus in control and *Cnot3* CKO embryos harboring the *NDE-Hsp-dsVenus-*3′*-UTR* transgene at the four-somite stage. Note that Venus fluorescence is increased on the left side of the *Cnot3* CKO embryo. Scale bars, 50 µm. Images shown in (**d**), (**e**), and (**f**) are representative of thirteen, fourteen, and seven embryos, respectively. **g** Summary of the L-R patterns of *Dand5* mRNA expression. The *p* value of the difference between the indicated genotypes was determined using the two-sided Wilcoxon rank sum test. The numbers in bars indicate the number of embryos per genotype.

In *Drosophila*, an ortholog of Bicc1 regulates mRNA decay by recruiting the Ccr4-Not complex through direct binding to its Not3/5 subunit^15^. Although Bicc1-mediated mRNA decay has not been observed previously in vertebrates^11,27^, several Ccr4-Not subunits were also found to be enriched by mouse Bicc1 in a protein interactome screen in T-Rex HEK293 cells^28^. Consistent with a possible interaction in vivo, immunofluorescent staining of endogenous Bicc1 and Cnot3 proteins in mouse embryos significantly overlapped in the cytoplasm of node crown cells (Fig. 8a). In good agreement, immunostaining of transfected Cnot3-FLAG overlapped with large cytoplasmic HA-Bicc1 foci at their periphery, and with polymerization mutant HA-Bicc1(mutD), which stains more diffusely due to a mutation in the SAM-SAM interface (Supplementary Fig. 10). To further test this interaction and whether it involves Bicc1 polymerization or RNA binding, we performed co-immunoprecipitation assays in HEK293T cells. Both endogenous Cnot1 and FLAG-tagged Cnot3 co-immunoprecipitated with HA-Bicc1, irrespective of prior treatment with RNase A (Fig. 8b and c). Moreover, while deletion of the Bicc1 KH repeats significantly diminished the co-immunoprecipitation of both Cnot3-Flag and of endogenous Cnot1, deletion of the SAM domain did not (in the case of Cnot1) or even increased it (in the case of Cnot3-FLAG). Besides corroborating the observed protein colocalization, these results suggest that mammalian Bicc1 recruits the CCR4-Not complex at least in part through its KH domains, independently of RNA and of the SAM domain.

**Fig. 9:**
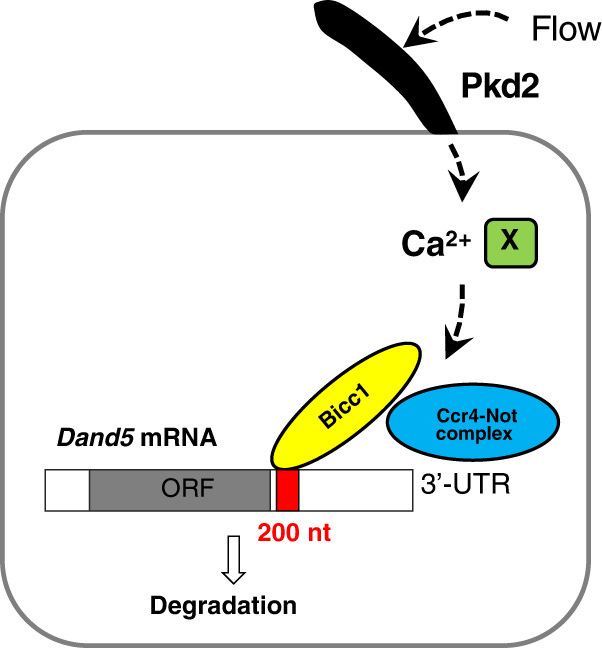
Model for the degradation of *Dand5* mRNA in crown cells of the node in response to nodal flow. See text for details.

To examine whether Cnot3 contributes to the decay of *Dand5* mRNA in crown cells at the node, we generated Cnot3 conditional knockout (CKO) embryos by crossing a *Cnot3*^flox/flox^ mouse^29^ with a *Noto*^CreERT2/+^ mouse^30^. The latter expresses Cre recombinase in a tamoxifen-inducible manner specifically at the node beginning at E8.0. Control (*Cnot3*^flox/flox^;*Noto*^+/+^, *Cnot3*^flox/+^;*Noto*^+/+^, *Cnot3*^+/+^;*Noto*^CreERT2/+^ and *Cnot3*^+/+^;*Noto*^CreERT2/CreERT2^) and CKO (*Cnot3*^flox/flox^;*Noto*^CreERT2/+ or CreERT2/CreERT2^) embryos isolated from tamoxifen-treated dams were first examined for *Nodal* expression. Nodal expression was maintained at the node and in LPM in all the control embryos examined (n = 8/8)(Fig. 8d). In about a half of *Cnot3* CKO embryos (*n* = 7/13), *Nodal* mRNA was maintained at the node but was lost in LPM (Fig. 8d), consistent with the possibility that the stability of *Dand5* mRNA in crown cells was increased. We then examined *Dand5* expression in control and CKO embryos at the four- to six-somite stages. L-R asymmetry of *Dand5* mRNA was lost (*n* = 6/14) or attenuated (*n* = 6/14) in the CKO embryos Fig. 8e, g). Similarly, L-R asymmetry of the 3′-UTR reporter transgene was lost or attenuated in the CKO embryos (*n* = 7/7) (Fig. 8f, and Supplementary Fig. 1d). When *Cnot3* CKO and control embryos were examined at a later stage (E9.5), some of the CKO embryos (2/25 embryos) exhibited heart looping defects whereas none of the control embryos (0/25 embryos) showed such heart defects (Supplementary Fig. 11). The low frequency of the heart defects in the CKO embryos is likely due to the incomplete deletion by *Noto*^*CreERT2*^ and the robustness of the asymmetry-generating system that operates at the node and in the LPM^24,31^. These results suggest that the Ccr4-Not complex in crown cells indeed plays a role in the degradation of *Dand5* mRNA in response to nodal flow (Fig. 9).

## Discussion

To delineate how fluid flow establishes left-right asymmetry in the mouse embryo, we have generated a dsVenus transgene that reports the decay of *Dand5* mRNA in crown cells of the node and its acceleration by directional fluid flow and by Pkd2 specifically on the left side. Our data show that a proximal 3′-UTR fragment mediating asymmetric *Dand5* mRNA expression binds and genetically interacts with the RNA binding protein Bicc1 through a newly identified Bicc1-binding element comprising two GAC motifs that are juxtaposed within the octameric sequence GACGUGAC. In turn, Bicc1 binds to the Ccr4-Not deadenylase complex which mediates increased degradation of the *Dand5* mRNA in response to Pkd2 stimulation by leftward fluid flow.

Bicc1 binds to various target mRNAs, but no consensus motif recognized by this protein has been identified. Our comparison of various mammalian *Dand5* genes revealed that conservation among the 3′-UTR sequences is largely limited to the 200-nucleotide proximal-most region which we found to be required for the establishment of L-R asymmetry of *Dand5* mRNA in crown cells. The only conserved features in this region or elsewhere in the 3′-UTR among mammalian *Dand5* genes consist of a predicted stem-loop with a GACGUGAC motif at its base, and enrichment of additional GAC motifs. Using EMSA and co-immunoprecipitation assays, we found that the GACGUGAC sequence mediates specific binding to Bicc1. Since KH domains accommodate single-stranded 4-nucleotide sequences^23^, and since our RBNS analysis in vitro revealed a preference of Bicc1 for GACR and YGAC motifs, Bicc1 binding to GACGUGAC in the *Dand5* 3′-UTR where these motifs are juxtaposed likely involves at least two KH domains. While GACG was essential for specific Bicc1 binding in vitro, UGAC further increased this interaction. Conversely, we found that a mutation in the KH2 domain blocked binding to the *Dand5 3*′*-UTR* in HEK293T cells, in line with recent findings on *Cripto1* mRNA binding in *Xenopus*^32^. Since maximal binding to the *Dand5 3*′-UTR also required the KH1 domain, KH2 and the GACG motif in *Dand5* mRNA may be essential to prime a complex that is then stabilized by KH1 binding to the adjacent UGAC sequence. In line with this model, at least the GACG motif should be accessible, whereas the UGAC motif resides in the stem of a predicted stem-loop (Fig. 7a). Moreover, in the Bicc1-related *C. elegans* protein GLD-3, the KH1 domain is structurally independent, whereas the other KH domains are interconnected through extended β-sheets to form a rigid platform^33^. Thus, the KH1 domain may be flexible enough to pry open the UGAC motif within the stem once the necessary entropy cost is diminished by prior binding of GACG to KH2 (Supplementary Fig. 9). By a related mechanism, one continuous surface of two KH domains in the bacterial protein NusA can melt a stem-loop in associated RNA to accommodate an extended sequence of 11 nucleotides^34^. The apparent conservation of the consensus sequence GACN_1-2_GAC in mammalian *Dand5 3*′*-UTRs* suggests that Bicc1 may recognize this extended motif, with a possible contribution of the proximal poly-purine tract R_3-4_ and a central linker N_1-2_ of variable length that could be adapted to species-specific constraints in Bicc1. Indeed, similar extended motifs of two GAC sequences preceded by a poly-purine tract are conserved among mammals and in *Xenopus* (Fig. 6h), where they are part of a  36-nucleotide region that is required for flow-induced decay of the *Xenopus Dand5S* mRNA (Maerker et al., accompanying paper)^21^. However, these findings do not exclude the possibility that Bicc1 may diversify its panel of target transcripts by using different combinations of its three KH domains to bind alternative combinations of GAC motifs.

Bicc1 binds to various target mRNAs. Bicc1 is expressed not only in peri-nodal cells but also in pit cells with motile cilia (Fig. 4). In about a half of *Bicc1*^Δex1/Δex1^ embryos, motile cilia at the node exhibited polarity defects (Supplementary Fig. 4), suggesting that Bicc1 has additional target mRNAs in pit cells. On other target mRNAs such as mouse *Adcy6* or *Xenopus Cripto1*, Bicc1 has been shown to inhibit their translation^11,27,35^. In crown cells of the mouse node as well as in ciliated cells at the gastrocoel roof plate of *Xenopus* embryos, however, the level of *Dand5* mRNA eventually becomes asymmetric (R > L) in response to nodal flow^9^, suggestive of increased mRNA decay. Bicc1-mediated regulation of certain mRNAs has been shown to involve microRNAs (miRNAs)^11,36^, and miRNA-mediated inhibition of target mRNAs typically involves both translational repression and mRNA decay^37^ mediated by Ccr4-Not^38^. Consistent with a potential role for miRNAs in the regulation of *Dand5* mRNA in crown cells of the mouse embryo, deletion of *Dicer* in these cells abolished asymmetric *Nodal* expression in the lateral plate (Maerker et al., accompanying paper)^21^. However, given that no miRNA seed sequences are highly conserved in the 3′-UTR of mammalian *Dand5* mRNAs (Fig. 3a) and that Ccr4-Not can also be recruited by specific RNA-binding proteins independently of miRNAs^39^, the regulation of *Dand5* mRNA by Bicc1 may be mediated by an miRNA-independent pathway. In all, our data indicate that inhibition of Dand5 expression at the mouse node is dependent on the 3′-UTR of the mRNA and mediated by Bicc1 and Cnot3 at the level of mRNA decay.

The degradation of *Dand5* mRNA in crown cells occurs predominantly on the left side of the mouse node. How can the Bicc1-Cnot3 pathway be activated on the left side but not on the right side? Several lines of evidence implicate Ca^2+^ in the decay of *Dand5* mRNA. First, the Dand5-dependent asymmetry (R < L) of Nodal activity in crown cells is disrupted by various Ca^2+^ blockers including thapsigargin^5^ and ionophore^40^. Furthermore, R > L asymmetric expression of the *NDE-Hsp-dsVenu’-3*′*-UTR* transgene at the node in the present study was impaired by thapsigargin treatment and by the absence of Pkd2. The decay of *Dand5* mRNA may therefore be induced by the Ca^2+^ influx triggered by sensing of nodal flow by the immotile cilia of crown cells^6^. The resulting increase in the cytosolic concentration of Ca^2+^ may influence, directly or indirectly, either the interaction with a component (X in Fig. 9) of the pathway responsible for mRNA decay (such as between Bicc1 and *Dand5* mRNA, or between Bicc1 and Ccr4-Not) or the deadenylase activity of Ccr4-Not. Of note in this regard, crown cells at the mouse node express calmodulin and proteins such as Inversin with a calmodulin-binding motif, some of which are known to be essential for L-R asymmetry^41,42^. Future studies are thus warranted to probe further the role of Ca^2+^ in the regulation of *Dand5* mRNA decay.

## Methods

### Mouse strains

All mouse experiments were performed in accordance with guidelines of the RIKEN Center for Biosystems Dynamics Research and under an institutional license (A2016-01-6). Mice were maintained in the animal facility of the RIKEN Center for Biosystems Dynamics Research. *Noto-Cre*^ERT2^ mice^30^, *Cnot3*^flox/+^ mice^29^, *iv* mice^19^, and *Pkd2* mutant mice^3^ were described previously. Expression of the *Noto-Cre*^ERT2^ transgene in embryos was induced by oral administration of tamoxifen (Sigma) in corn oil to pregnant mice at a dose of 5 mg both 24 and 12 h before the late headfold stage.

### Cell lines

HEK293T and HeLa cells were cultured in DMEM (Sigma) supplemented with 10% FBS (Sigma), 1% GlutaMAX (Thermo Fisher Scientific), and 1% gentamicin (Thermo Fisher Scientific). The cells were transfected with expression plasmids with the use of jetPEI (Polyplus Transfection). IMCD cells were cultured in DMED/F12 (1:1) (Gibco 11320-033) with 10% FBS. For RBNS analysis, the human embryonic kidney cell line 293FT (Invitrogen, #R70007) was cultured under a humidified atmosphere of 5% CO_2_ at 37 °C in Dulbecco’s modified Eagle’s medium (DMEM) (Nacalai tesque, #08459-64) supplemented with 10% fetal bovine serum (FBS) (Biosera, #554-02155, lot #10259), 2 mM L-glutamine (Thermo Fisher Scientific, #25030-081), 1 × MEM Nonessential Amino Acids (Thermo Fisher Scientific, #11140-050), and 1 mM sodium pyruvate (Sigma, #S8636-100ML).

### Transgene design

To generate the transgene *NDE-Hsp-dsVenus-3*′*-UTR* that confers expression of dsVenus at the node of mouse embryos, the dsVenus coding sequence was linked to the 1.2 kb DNA sequence for the 3′-UTR of mouse *Dand5* mRNA and placed under the control of four copies of the crown cell-specific enhancer (NDE) of the mouse *Nodal* gene and the mouse *Hsp68* promoter. Transgenes containing only the proximal 200 bp region (200prox) or a truncated 3′-UTR lacking the proximal or distal 200 bp region (NDE-Hsp-dsVenus-3′-UTRΔ200prox or Δ200dist) were generated analogously.

### Generation of transgenic and mutant mice

*Dand5*^Δ200prox/Δ200prox^ embryos (Fig. S1) and *Bicc1*^Δex1/ Δex1^ embryos (Fig. S2) were generated with the use of the CRISPR/Cas9 system. For the generation of transgenic mice, each transgene was microinjected into the pronucleus of fertilized eggs obtained by crossing C57BL/6 J female and male mice. The eggs were then transferred to pseudopregnant mothers. In some instances (embryos shown in Fig. 3B, D, E), transgenic embryos were directly examined before the establishment of stable transgenic lines. *Dand5* and *Bicc1* mutant mice and embryos were genotyped by genomic PCR analysis with the primers indicated in Supplementary Figs. 3 and 4.

### Plasmids, cloning, and mutagenesis

The plasmid pCMV6-Entry Cnot3-Myc-FLAG encoding mouse Cnot3 was obtained from Origene (MR210463). The pCMV-SPORT6 HA-Bicc1 plasmids for WT, ΔKH, ΔSAM, and mutD forms of mouse Bicc1 were previously described^10,12^. Vectors encoding mutKH1, mutKH2, mutKH3, or mutKH1,2,3 were generated by overlap extension PCR and subcloned between the SalI and BstBI sites of pCMV-SPORT6 HA-Bicc1. Vectors encoding the *Dand5 3*′*-UTR* variants mutU and Stem+ were generated by overlap extension PCR and subcloned at the unique NotI site of pEFBOS *Dand5-3*′*UTR(200prox)*. The sequences of all mutated constructs were verified by Sanger sequencing.

### Recombinant protein production

Recombinant GST-Bicc1-KH was were expressed in *E. coli* BL21(DE3)pLysS cells (Promega) and purified by affinity chromatography on glutathione–sepharose beads (GE Healthcare) in 50 mM Tris-HCl (pH 7.5), 200 mM NaCl, 1 mM DTT. After elution in 50 mM Tris-HCl (pH 8), 150 mM NaCl, 10 mM reduced glutathione, 1 mM DTT, the protein was dialyzed and concentrated on Amicon Ultra-0.5 mL column (Merck, UFC500396).

### WISH analysis

WISH was performed according to standard procedures with digoxigenin-labeled riboprobes specific for *Dand5*, *Nodal*, and *dsVenus* mRNAs^43^.

### Immunofluorescence analysis

Dissected embryos were fixed with 4% paraformaldehyde, dehydrated with methanol, and permeabilized with phosphate-buffered saline (PBS) containing 0.1% Triton X-100. They were incubated overnight at 4 °C with primary antibodies at the following dilutions: anti-Bicc1 (1:100 dilution, rabbit polyclonal, Sigma), anti-acetylated tubulin (1:100 dilution, mouse monoclonal, Sigma), anti-ZO1 (1:10 dilution, mouse monoclonal, clone ZO1-1A12, Invitrogen), anti-Cnot3 (mouse monoclonal, 1:50–100 dilution, described previously^16^) and anti-Odf2 (1:100 dilution, rabbit polyclonal, Abcam). The embryos were washed with PBS containing 0.1% Triton X-100 before exposure to Alexa Fluor-conjugated secondary antibodies (Invitrogen) and DAPI (Wako). The node of stained embryos was excised, placed on a slide glass with silicone rubber spacers, covered with a cover glass, and imaged with an Olympus FV1000 or FV3000 confocal microscope. To localize Bicc1 and Cnot3 in node cells, cryosections were prepared from immunostained node samples and imaged by super-resolution AiryScan mode using an LSM880 confocal microscope (Zeiss). For immunostaining of HeLa cells, cells cultured in six-well plates and transfected with 1 µg of each expression vector for 24 h were transferred to sterile coverslips in the wells of a 24-well plate. After culture for an additional 24 h, the cells were fixed for 10 min at −20 °C in methanol, washed with PBS, and incubated at room temperature first for 1 h in PBS containing 1% bovine serum albumin as a blocking agent and then for 2 h in the blocking solution containing antibodies to HA (1:500, rabbit monoclonal, Sigma) and to FLAG (1:500, mouse monoclonal clone M2, Sigma). After washing with PBS, the cells were incubated for 1 h at room temperature with Alexa Fluor 568- or Alexa Fluor 647-conjugated secondary antibodies to mouse or rabbit immunoglobulin G, respectively, in blocking buffer containing DAPI (1:10,000 dilution). Images were acquired with a Zeiss LSM700 confocal microscope and deconvoluted using Huygens Remote Manager (https://hrm-biop.epfl.ch/).

### Observation of nodal flow by PIV analysis

Nodal flow was observed by multipoint scanning confocal microscopy. Particle image velocimetry (PIV) analysis was performed as described previously^18^. Recovered embryos were first cultured under 5% CO_2_ for 30 min at 37 °C in DMEM supplemented with 75% rat serum. The region containing the node was then excised, and the node cavity was filled with DMEM supplemented with 10% FBS and 0.2 µm-diameter fluorescent microbeads (Invitrogen, F8811). The motion of the beads was monitored for 10 s in planes of +5 and +10 µm relative to the cavity (21 frames per second) with the use of a CSU-W1 confocal unit (Yokogawa) and an iXon-Ultra EMCCD camera (Andor Technology) connected to an IX83 microscope (Olympus) fitted with a ×60 objective lens. Time-series images for PIV analysis were captured at a resolution of 512 by 512 pixels and were processed with interrogation windows of 16 by 16 pixels with 50% overlap, corresponding to a spatial resolution of 4.3 by 4.3 mm. The time-averaged velocity distributions were calculated for 10-s intervals.

### Quantitative analysis of basal body position

The average basal body position (ABP) representing the relative position of the basal body in each node cell along the A–P axis (vertical) was analyzed as described previously^44,45^. Briefly, confocal images of the node stained with antibodies to Odf2 and to ZO-1 were obtained to determine the position of the basal body in each node cell. For characterization of the shape and orientation of the node cells, the outline of each cell was calculated from the pattern of ZO-1 staining with the use of an ImageJ software plugin (http://rsb.info.nih.gov/nih-image) to apply watershed segmentation. The basal body was traced manually according to the Odf2 staining image, and the *x* and *y* coordinates of each basal body were recorded with the use of the graphical user interface (GUI) of MATLAB software. The relative value for the position of the basal body in each cell was calculated from the coordinate data with the anterior and posterior ends of the cell expressed as −1.0 and +1.0, respectively.

### Mouse embryo culture

Embryos were cultured under conditions of artificial fluid flow as described previously^20^. Briefly, embryos were collected at E7.5. Those at the early headfold stage were selected and cultured for 1 h with rotation under 5% CO_2_ at 37 °C in DMEM supplemented with 75% rat serum. They were transferred to a flow chamber, were cultured in DMEM with 50% rat serum for 7 h, and were then subjected to conventional rotation culture for additional ~2 h. Where indicated, thapsigargin was added to the medium at a final concentration of 500 nM for 1 h (during the initial rotation culture) and the embryos were then cultured in the absence of the drug. The node cavity was imaged with an Olympus FV1000 confocal microscope.

### Imaging and analysis of dsVenus

The node of *NDE4-hsp-dsVenus-Dand5-3*′*-UTR* transgenic embryos was excised in DMEM supplemented with 75% rat serum, placed on glass slides with silicone rubber spacers and glass cover slips for imaging with an Olympus FV1000 confocal microscope. The sum of XYZ images was obtained with Fiji/ImageJ as a 32-bit image. ROIs were set so as to include all crown cells on the left or right side of the node, and cells with fluorescence were selected. The mean signal intensity was measured for the left and right sides of the node, and the R/L ratio of the mean intensity was then determined.

### Protein co-immunoprecipitation and Western blot analysis

HEK293T cells in two 10-cm dishes per condition (10^7^ cells per dish) were transfected with expression plasmids for Cnot3-FLAG (2 µg) and either HA-Bicc1(WT) (2 µg), HA-Bicc1(ΔKH) (1 µg), HA-Bicc1(ΔSAM) (4 µg), or HA-Bicc1(mutD) (4 µg). After incubation for 3 days, cells from the two dishes for each condition were then washed with PBS and pooled in extraction buffer containing 20 mM Tris-HCl (pH 7.4), 2.5 mM MgCl_2_, 100 mM NaCl, 5% glycerol, 1 mM dithiothreitol (DTT), 0.05% Nonidet P-40 (NP-40), RNasin (Promega), phosphatase inhibitors (Sigma), and protease inhibitors (Roche). Total cell extracts were prepared by ultrasonication followed by two rounds of centrifugation at 10,000 × *g* for 5 min at 4 °C to remove debris. Supernatants were incubated with 20 µl of anti-HA beads (Sigma) for 2.5 h at 4 °C on a rotating wheel. For RNase treatment, cell extracts were supplemented with 12.5 µg/mL of RNase A (Roche) and incubated for 10 min at RT prior to incubation on beads. The beads were rinsed four times for 10 min in wash buffer (20 mM Tris-HCl pH 7.4, 2 mM MgCl_2_, 200 mM NaCl, 1 mM DTT, and 0.1% NP-40), suspended in Laemmli buffer, and directly loaded on reducing SDS PAGE gels to size-separate eluted proteins. For immunoblot analysis, proteins were transferred to nitrocellulose membranes, blocked with skim milk, and incubated overnight with the antibodies indicated, and with fluorescently labeled secondary antibodies for analysis on a Odyssey CLx scanner (LI-COR Biosciences). The signal of co-immunoprecipitated protein was normalized relative to the signal of Bicc1 in the IP fraction.

### RNA co-immunoprecipitation in HEK293T cells

HEK293T cells were transfected as described above, but with an expression plasmid for the ORF of dsVenus linked to the WT or Δ*200* forms of the 3′-UTR sequence of mouse *Dand5* mRNA (2 µg per dish) together with a plasmid encoding WT or ΔKH mutant forms of HA-Bicc1 (2 or 1 µg per dish, respectively). Cytoplasmic extracts prepared after 36 h as described above were passaged eight times through a syringe needle (no. 30) followed by centrifugation twice at 10,000 × *g* for 5 min at 4 °C. After setting aside 5% of each supernatant as input, the remainder was immunoprecipitated as described above. While a portion of the beads (10%) was analyzed by immunoblotting as described above, the remainder of the beads and half of the input samples were subjected to phenol-chloroform extraction. After ethanol precipitation isolated RNA was treated with RQ1 DNase (Promega) and converted to cDNA by PrimeScript Reverse Transcriptase Kit (Takara). The resulting cDNA was then subjected to PCR or qPCR using Phire Green Hot Start II PCR Master Mix (Promega) or GoTaq qPCR Master Mix (Promega), respectively, with the following primers: F1 (GCTGAGCATCCTAGAGGAATGC), F2 (TGCCACAATCACTAACTCACGTC, R1 (TGTCTTGGACACTGGGACGC) and R2 (TAAACCCATGACTGGGGGACCATGTCTAG) for the 3′-UTR of *Dand5* mRNA, and ACAGAGCCTCGCCTTTGCC and CTCCATGCCCAGGAAGGAAGG (forward and reverse, respectively) for β-actin mRNA. The amount of co-immunoprecipitated mRNA as a percentage of the input was calculated with the formula: 100 × 2[(Ct(Input) − log_2_(100/2.5) − Ct(IP)], where the value 2.5 represents 2.5% of the original cytoplasmic extract, and where Ct is the cycle threshold for qPCRs on input or immunoprecipitate (IP) samples. Fold enrichment was calculated relative to cells transfected with the corresponding empty vector for HA-Bicc1.

### RBNS analysis

RBNS analysis was performed essentially as described by others^26^. A double-stranded DNA template for in vitro transcription of a 20-mer random RNA library was synthesized with a primer extension reaction in which 100 µl of a reaction mixture containing 1 × Platinum SuperFi PCR Master Mix (Thermo Fisher Scientific, #12358-010), 1× SuperFi GC Enhancer (Thermo Fisher Scientific), 100 nM DNA template oligomer (5′-GAAATTAATACGACTCACTATAGGACGTGACACGACGTGCGCN_20_GCGTACGTCGGACCTCAGGTCGACCATGGACGC-3′, where N_20_ is the DNA sequence encoding the 20-mer RNA sequence), 100 nM primer (5′-GCGTCCATGGTCGACCTGAGGTCC-3′), and nuclease-free water was incubated at 98 °C for 130 s, at 50 °C for 2 min, and then at 72 °C for 10 min. The synthesized DNA template was purified with the use of a Monarch PCR & DNA Cleanup Kit (New England Biolabs, #T1030L). The random RNA library was then transcribed with the use of a MEGAshortscript T7 Transcription Kit (Thermo Fisher Scientific, #AM1354) in a reaction mixture containing 1 × Reaction Buffer, 1 × T7 Enzyme Mix, 7.5 mM ATP, 7.5 mM UTP, 7.5 mM GTP, 7.5 mM CTP, and 9.15 pmol of the DNA template. The mixture was incubated at 37 °C for 6 h, after which the DNA template was digested with TURBO DNase for 30 min at 37 °C and the transcribed RNA library (5′-GGACGUGACACGACGUGCGCN_20_GCGUACGUCGGACCUCAGGUCGACCAUGGACGC-3′) was purified with an RNA Clean & Concentrator (Zymo Research, #R1016).

For generation of an expression vector for Bicc1-FLAG (pcDNA3.1-Bicc1-FLAG), the Bicc1 ORF was first amplified from Bicc1/mBS and then inserted between the BamHI and NotI sites of pcDNA3.1/myc-His A (Invitrogen, #V80020) to generate pcDNA3.1-Bicc1-myc-His A. The sequences corresponding to the c-Myc and His tags in pcDNA3.1-Bicc1-myc-His A were replaced with that for the FLAG tag by PCR-based mutagenesis, thereby generating pcDNA3.1-Bicc1-FLAG. 293FT cells (3 × 10^6^ cells) were seeded in a 10-cm cell culture dish and cultured for 24 h before transfection for 24 h with 15 µg of pcDNA3.1-Bicc1-FLAG with the use of Lipofectamine 3000 (Thermo Fisher Scientific, #L3000008) according to the manufacturer’s instructions. As a mock transfection control, cells were treated with Lipofectamine 3000 alone. The cells were then collected by centrifugation at 300 x *g* for 5 min at 4 °C, washed with ice-cold PBS, and lysed by incubation for 30 min on ice, with intermittent vortex mixing, in 1 ml of Cell Lysis Buffer (Invitrogen, #FNN0021) supplemented with 1 × complete Protease Inhibitor Cocktail (Roche, #04693116001) and 1 mM phenylmethylsulfonyl fluoride (Thermo Fisher Scientific, #36978). The cell lysates were centrifuged at 15,300 × g for 10 min at 4 °C, and the resulting supernatants were collected, assayed for protein concentration with the use of a Pierce BCA Protein Assay Kit (Thermo Fisher Scientific, #23227), adjusted to a protein concentration of 700 µg/ml with cell lysis buffer, and stored at −80 °C.

The cell lysate containing Bicc1-FLAG was diluted fourfold with the mock cell lysate for immunoprecipitation. For preparation of antibody-coated magnetic beads, 50 µl of Dynabeads Protein G (Thermo Fisher Scientific, #DB10003) were conjugated with 10 µg of M2 mouse monoclonal antibodies to FLAG (Sigma, #F1804) according to the manufacturer’s instructions. The antibody-coated beads were suspended in 400 µl of Ab Binding & Washing Buffer [PBS containing 0.02% Tween-20 (Roche, #11332465001)] and mixed with 400 µl of the cell lysate. After incubation for 1 h at 4 °C with rotation, the beads were separated with a magnet and the supernatant removed. The beads were washed with 1 ml of RNP Binding Buffer [20 mM Tris-HCl (pH 7.4), 2.5 mM MgCl_2_, 80 mM NaCl, 20 mM KCl, 5% glycerol (Sigma, #G5516-100ML), 1 mM DTT (Thermo Fisher Scientific, #A39255), 0.05% NP-40 (Thermo Fisher Scientific, #85124)], suspended in 500 µl of RNP Binding Buffer containing 500 pmol of the random RNA library, incubated for 16 h at 4 °C with rotation, isolated with the magnet, and washed five times with 1 ml of RNP Binding Buffer. The beads were then incubated for 3 min at 95 °C in 200 µl of an elution buffer (1% SDS, 10 mM Tris-HCl, and 2 mM EDTA) before separation with the magnet, and the supernatant was collected. The eluted RNA was isolated by phenol-chloroform extraction and ethanol precipitation. The RNA pellet was suspended in 24 µl of nuclease-free water and subjected to RT with SuperScript IV Reverse Transcriptase (Thermo Fisher Scientific, #18090010). Prior to reverse transcription, 20 µL of purified RNA library was mixed with 1 µL of 10 µM reverse primer (5′-GCGTCCATGGTCGACCTGAGGTCC-3′), 1 µL of 10 mM dNTPs, and 4 µL of nuclease water, and then denatured at 65 °C for 5 min. The mixture was combined with a premix of 8 µL of 5X SSIV Buffer, 2 µL of 100 mM DTT, 2 µL of RNase OUT (Thermo Fisher Scientific, #10777019), and 2 µL of SuperScript IV Reverse Transcriptase. The mixture was incubated at 50°C for 10 min, then at 85 °C for 10 min. To prepare an input random RNA library, 0.5 pmol of input random RNA library was also reverse transcribed. After digestion of RNA with 2 µl of RNaseH for 20 min at 37 °C, the cDNA preparation was amplified by PCR in a reaction mixture containing 1× Platinum SuperFi PCR Master Mix, 5 µM forward primer (5′-TCGTCGGCAGCGTCAGATGTGTATAAGAGACAGGGACGTGACACGACGTGC-3′), 5 µM reverse primer (5′-GTCTCGTGGGCTCGGAGATGTGTATAAGAGACAGGGCGTCCATGGTCGACCTGAGGTCCGACG-3′), 1 × SuperFi GC Enhancer, and 2 µl of cDNA and adjusted to 25 µl with nuclease-free water. The incubation protocol included an initial denaturation at 98 °C for 30 s, 20 cycles of denaturation at 98 °C for 10 s and extension at 72 °C for 10 s, and a final extension at 72 °C for 5 min. Single-stranded DNA was digested with Illustra ExoProStar exonuclease I (GE Healthcare Life Sciences, #US78211), and the PCR products were purified with the use of a MinElute PCR Purification Kit (Qiagen, #28006) before the addition of Illumina sequencing barcodes by 10 cycles of PCR with index primers from a Nextera XT Index Kit (Illumina, #FC-131-1001). The reaction mixture contained 1× Platinum SuperFi PCR Master Mix, 5 µl of Index 1 (i7) primer, 5 µl of Index 2 (i5) primer, 1 × SuperFi GC Enhancer, and at most 5 µl of the PCR products, and the volume was adjusted to 50 µl with nuclease-free water. The incubation protocol included an initial denaturation at 98 °C for 30 s; 10 cycles of denaturation at 98 °C for 10 s, annealing at 55 °C for 10 s, and extension at 72 °C for 20 s; and a final extension at 72 °C for 5 min. The resulting sequencing library was quantified with the use of a KAPA Library Quantification Kit (Illumina) and Universal qPCR Mix (Kapa Biosystems, #07960140001). The library was sequenced for 150 cycles on the Illumina MiSeq platform and with a MiSeq Reagent Kit v3 (Illumina, #MS-102-3001). FASTQ files were processed as follows. The adapter sequences of the reads were trimmed by cutadapt 1.10 with parameter −e set to 0.2. Low-quality reads (averaged quality score of <30) were filtered with a custom script written in Julia 1.1. The filtered reads were split into overlapping k-mers (*k* = 4, 5, or 6); for example, the 20-mer sequence yielded 15 hexamers. The frequency of each k-mer was determined and normalized by the sum of all frequencies. With *k* denoting each k-mer, the following formula defines relative frequency: relative frequency(*k*) = normalized frequency(*k*)_Bicc1_/normalized frequency(*k*) _Control_. To identify Bicc1-bound sequence elements, we collected the hexamers whose *Z*-score for the relative frequency was >3.0. The enriched hexamers were aligned by Clustal Omega^46^ with default parameters. The motif logo was generated from the aligned hexamers by Weblogo3^47^.

### Metagene analysis

Mouse 3′-UTR sequences (mm10) were obtained from the UCSC Table Browser by specifying the track as ALL GENCODE V22 and the table as Basic. The 200-nt proximal regions were extracted, and duplicated sequences were removed. The number of motifs present in each region was then counted.

### Multiple alignment

Sequences were aligned by MAFFT^48^ or MAFFT at the MPI Bioinformatics Toolkit^49^ with manual changes to align positions of GAC motifs among species. Multiple alignment and folding of RNA were performed by LocARNA^50^.

### In vitro transcription

The plasmid pEFSA *Dand5-*3′UTR was used as a PCR template to generate SP6 transcription matrices. Briefly, a forward primer containing the SP6 promoter sequence and a Reverse primer were used for the amplification of the DNA fragment encoding the sequence of interest using the Phire Green Hot Start II PCR Master Mix (ThermoFisher F126L). For future annealing of a fluorescent probe, the complementary 5′-TGTCTGGGCAACAGGCTCAGG-3′ sequence was introduced as a 3′ tag via the Reverse primer. For the short transcripts *Dand5 3*′*UTR*_*66-110*_ and *Dand5 3*′*UTR*_*226-270*_, the transcription matrices were prepared without plasmid template using overlapping PCR primers carrying the mutations of interest. After agarose gel electrophoresis, PCR amplicons were purified on NucleoSpin® Gel and PCR Clean-up (Macherey-Nagel, Cat.No. 740609). 500 ng of SP6 transcription matrice was used to in vitro transcribe the RNA of interest by using the SP6 RNA polymerase kit according to the manufacturer’s conditions (Roche, Cat.No. 10 810 274 001). After 2 hours of incubation at 37 °C, the transcripts were incubated for one additional hour in presence of DNAse I (Roche, Cat.No. 04 716 728 001) in order to eliminate the SP6 DNA matrix. Finally, the transcripts were purified on Quick Spin Columns (Roche, Cat.No. 11 274 015 001).

### Fluorescent EMSA

The fluorescent DNA probe 5′-CTGAGCCTGTTGCCCAGAC-3′ carrying a 5′-Dynomics 681 dye, was synthesized by Microsynth AG. Before each experiment, 3′ tagged RNA and the fluorescent DNA probe were pre-annealed by denaturation (3 min at 98 °C) and renaturation for 10 min at room temperature. One pmol of 3′-tagged RNA and 2.5 pmol of fluorescent probe were mixed for each condition. Complexes of fluorescent RNA:DNA duplex with recombinant GST-KH were assembled in a final volume of 20 μL containing 10 mM Tris-HCl pH8, 100 mM KCl, 2.5 mM MgCl2, 2.5% glycerol, 1 mM DTT, and 1 µg of yeast tRNAs by incubation on ice for 30 min in the dark. For competition experiments, the recombinant GST-KH protein was used at a fixed concentration (200 nM). Increasing amounts of unlabeled competitor RNAs were added to pre-assembled fluorescent complexes and incubated for another 30 min. The complexes were then resolved by electrophoresis on a 5% native polyacrylamide gel containing 45 mM Tris-Borate, 1 mM EDTA, and 2.5% glycerol. The fluorescence was detected using the Odyssey CLx Infrared Imaging System (LI-COR Biosciences).

### Reporting summary

Further information on research design is available in the Nature Research Reporting Summary linked to this article.

## Supplementary information

Supplementary Information

Reporting Summary

## Data Availability

Sequencing data of this study were deposited to the Gene Expression Omnibus (GEO) database under accession number GSE140931. Publicly available data was downloaded from NCBI Data Base (https://www.ncbi.nlm.nih.gov), UCSC Table Browser (https://genome.ucsc.edu). Source data are provided with this paper.

## Source data

Source Data

## Code availability

Raw data for biochemical experiments are provided in Source data. The custom script of K-mer analysis is avaliable in the Github page (https://github.com/KRK13/Kmer2021). Other data and codes are available from the corresponding authors upon reasonable request.
