# Supplementary material for: Fluid flow-induced left-right asymmetric decay of Dand5 mRNA in the mouse embryo requires a Bicc1-Ccr4 RNA degradation complex
Source: Nat Commun. 2021 Jul 1;12:4071. doi: 10.1038/s41467-021-24295-2 (PMC8249388; doi:10.1038/s41467-021-24295-2)
Supplement: Supplementary file 1 — Supplementary Information [file 41467_2021_24295_MOESM1_ESM.pdf]

Supplementary Information

Fluid flow-induced left-right asymmetric decay of *Dand5* mRNA in the mouse embryo requires a Bicc1-Ccr4 RNA degradation complex

This file includes:
Supplementary Figs. 1 to 11

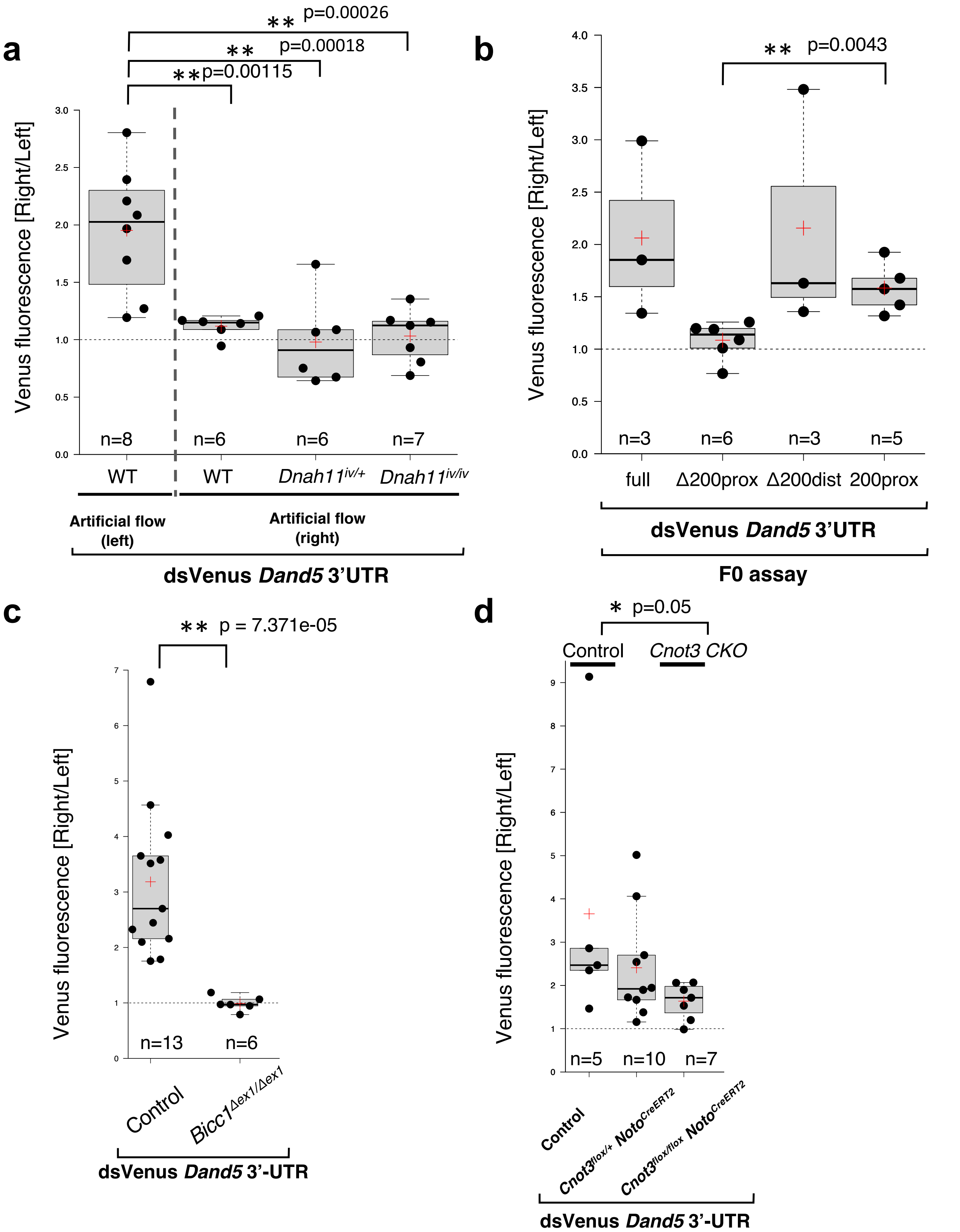

Supplementary Figure 1. Quantitative and statistical analysis of Venus expression by the *NDE-Hsp-dsVenus-3'-UTR* reporter transgene

a-d Right/Left ratios of Venus fluorescence at the node were quantified in groups of embryos of the indicated genotypes. The n values indicate the numbers of embryos analyzed, and red crosses indicate average values. For the boxplots, the top and bottom lines of each box represent the 75th and 25th percentiles of the samples, respectively. The line inside each box represents the median of the samples. The upper and lower lines above and below the boxes are the whiskers. Statistical significance (p) was determined by the two-sided Dunnett's test (**a**) or the two-sided Wilcoxon rank sum test (**b**)-(**d**). Source data are provided as a Source Data file.

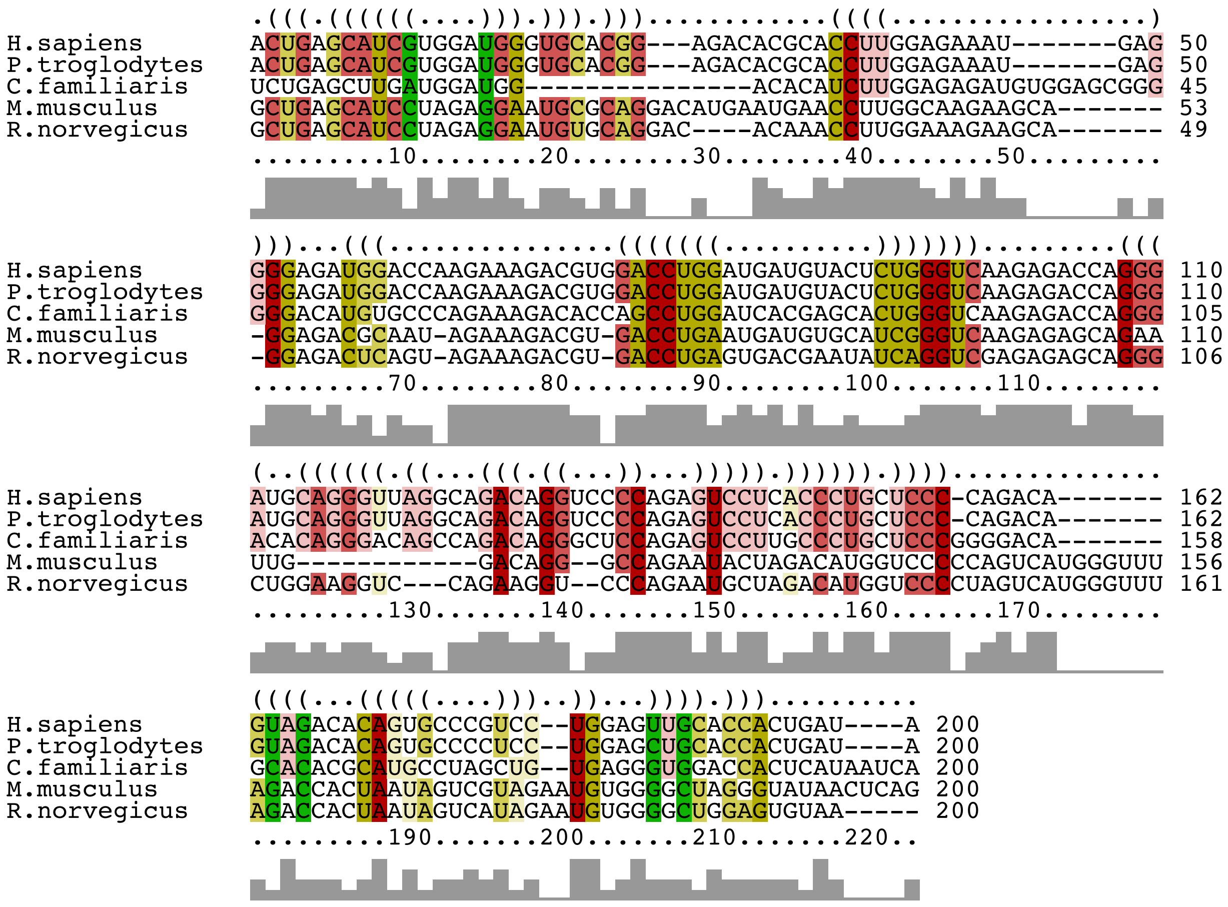

Supplementary Figure 2. Conserved RNA sequence and structure of the proximal 3'-UTR regions of Dand*5* mRNA

RNA sequences of the first 200 nucleotides in the 3’-UTRs of mammalian *Dand5* mRNAs were aligned using the using the LocARNA webserver . Predicted secondary structures are represented in dot-bracket format, and conserved base-pairings by the LocARNA color scheme.

a

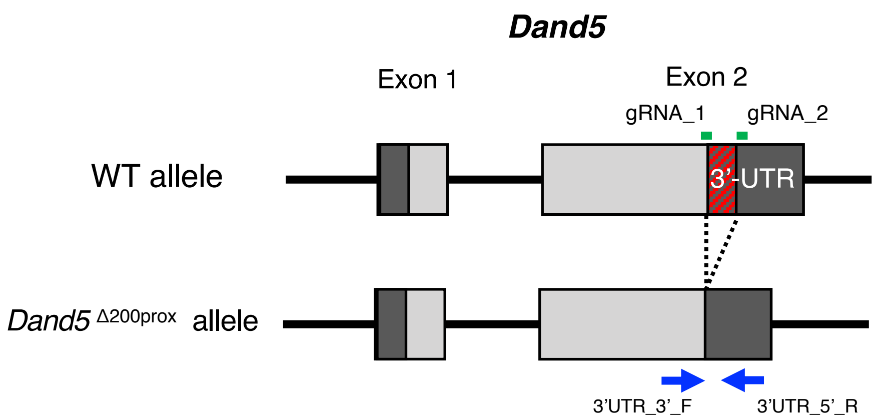

b

Exon 2 of *Dand5*

gtgatctccaggcctggttgcacaagtgcccgggtccttaatcatctctgttttggccgctgttcctccttctacattcccagctcg
gatcccacccctgtagtcttctgcaacagctgtgtgccggctcgaaagcgctggacatcggtgacgctgtggtgtggagctg
gccaattagcctcccctcggcgggtgaggatttccacggtattggtccagaagtgtcagtgccgc*ccgaagctgtgaGCT*
*GAGCATCC*TAGAGGAATGCGCAGGACATGAATGAACCTTGGCAAGAAGCAGGAGAC
GCAATAGAAAGACGTGACCTGAATGATGTGCATCGGGTCAAGAGAGCAGAATTGGA
CAGGGCCAGAATACTAGACATGGTCCCCCAGTCATGGGTTTAGACCACTAATAGTCG
TAGAATGTGGGGCTAGGGTATAACTCAGTGGGAGAGGGCTTGCCTAGCATGCAtgaag
*ccctgggttctattcgtatgtga*tatgtggaggttaaaaaaaggtgaaaattagctatagtgtatgattagccctgtgtgagagg
gaacattcatgccacaatcactaactcacgtcctcctcaggaatcctcagcagtcccgcaaggtagaatttcaccatgggtc
attttacagatgaggaaaccgaagctatttaggcactgtcacttttggagaggggtggaatagggagagggcttcagacac
agccctgacgcacgcgcagcacagctccctgctgagctcctcccccagcctggggttccgcttttagccactaggtggcac
cgcgtcccagtgtccaagacagacactcccaactgttatgatgacagcactacccaaacctggacactcagcaaagatga
ccctgttctggggacaccagaataacagcagcagcagcaacaacaacagcaacaaactatggcaggcaagcaggaa
gcagctgctaggaaaacctggccccaaccagaggcgaccccgcatgtgtgcagctgcctctctcatcgacagtagccag
gggtttcccttttgccccacccgagaccccgcagcagcctcatgcccagcctggcattccgtgggaaccataaaggttggc
caagtcc

3'-UTR
Red : Target sequence (gRNA) + PAM sequence
Expected deletion sequence

Target sequences
gRNA_1: ctgtgaGCTGAGCATCCTAGAGG
gRNA_2: tcacatacgaatagaacccaggg

c

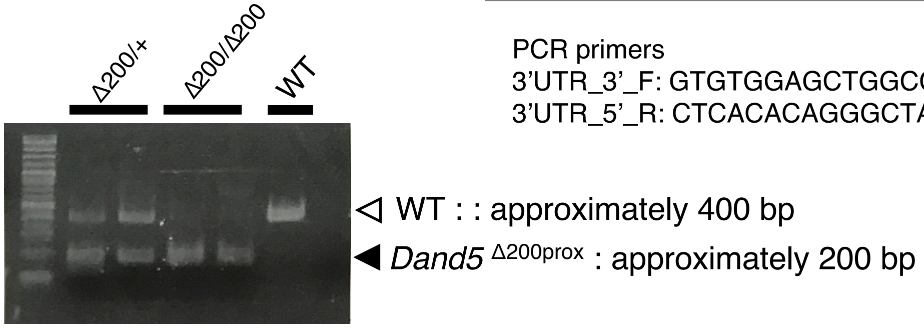

PCR primers
3'UTR_3'_F: GTGTGGAGCTGGCCAATTA
3'UTR_5'_R: CTCACACAGGGCTAATCATACA

Supplementary Figure 3. Generation of *Dand5*$^{\Delta 200prox}$ mutant mice using CRISPR/Cas9 editing

a Schematic representation of deletion of the 200-bp DNA sequence corresponding to the proximal-most region of the 3'-UTR of *Dand5* mRNA from the mouse genome. The red-shaded region of the WT allele was deleted to give rise to the *Dand5*$^{\Delta 200prox}$ allele. The positions of guide RNAs (gRNAs, green bars) and of PCR primers for genotyping (blue arrows) are indicated. **b** Nucleotide sequence of exon 2 of mouse *Dand5* showing portions related to deletion of the 200-bp fragment in (A) with the CRISPR/Cas9 system. **c** PCR-based genotyping of WT, *Dand5*$^{\Delta 200prox/+}$, and *Dand5*$^{\Delta 200prox/\Delta 200prox}$ mouse embryos with the indicated primers. Source data are provided as a Source Data file. Experiments were done at least twice with similar results.

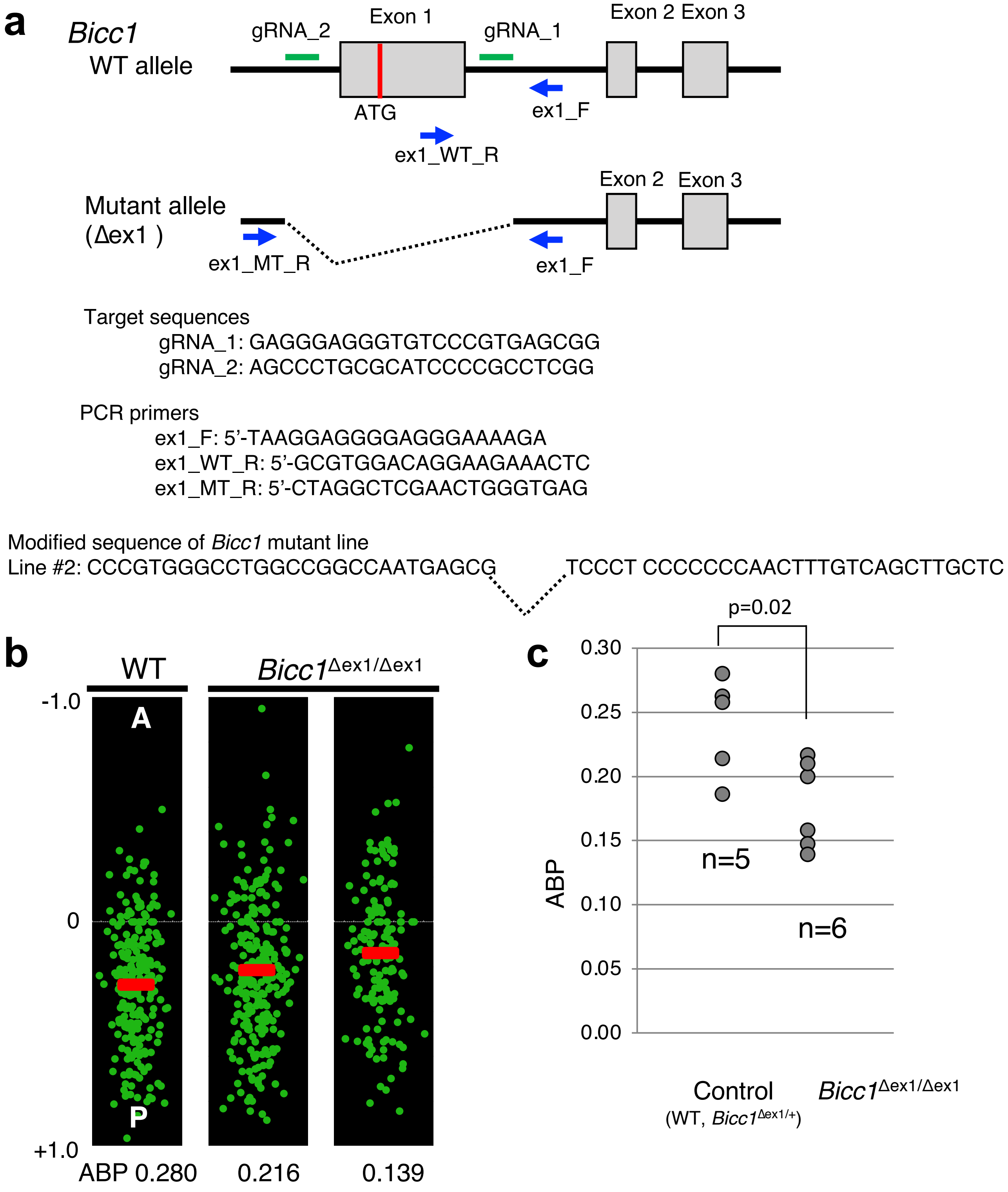

Supplementary Figure 4. Generation of *Bicc1* mutant mice by CRISPR/Cas9 editing
a *Bicc1* mutant mice were generated using two gRNAs (green bars) to delete exon 1. The positions of PCR primers (blue arrows) for genotyping are also indicated. **b** Relative position of the basal body in node cells of WT and *Bicc1*$^{\Delta ex1/\Delta ex1}$ embryos at three- to five-somite stages. Each green dot corresponds to the relative position of one basal body along the anterior (A)–posterior (P) axis. The average basal body position (ABP) for each embryo is indicated by the red bar, with the corresponding value being given below each image. **c** Summary of ABP for the indicated numbers of embryos (n) determined as in (b). The p value was determined using the two-sided Student's t-test. Source data are provided as a Source Data file.

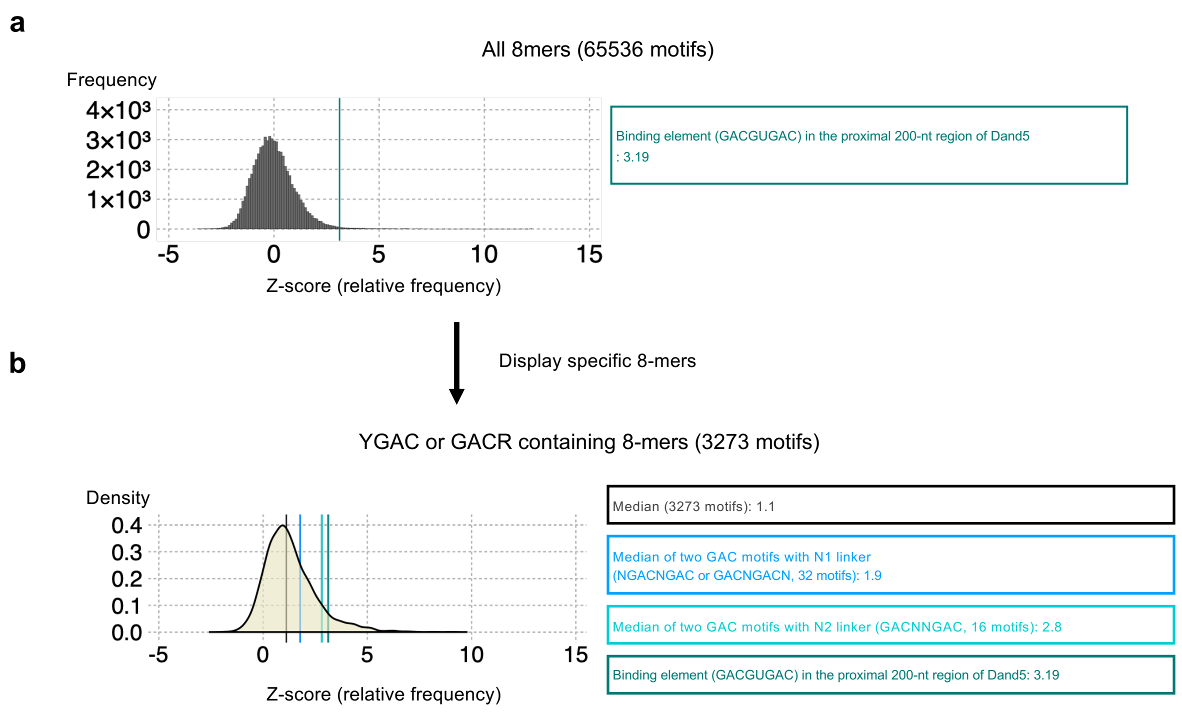

Supplementary Figure 5. Preferred sequence features of the Bicc1-Dand5 interaction
a RNA Bind-n-seq data analysis of counted 8-mers and their frequency distributions in Flag-Bicc1 immunoprecipitates. The enrichment score (Bicc1/control) of the Bicc1-binding element in mouse *Dand5 3'-UTR* is represented as a green line in the histogram. **b** Extracted 8-mers that include at least one YGAC or GACR motifs. In the histogram, colored lines indicate the median of YGAC- or GACR-containing 8-mers (black, 3273 motifs), the median Z-score of bipartite motifs with a single nucleotide linker (blue, 32 motifs), the median Z-score of bipartite motifs with dinucleotide linker (light green, 16 motifs), the Z-score of the validated binding element in the proximal 200-nt region of mouse *Dand5 3'-UTR* (green), respectively. The results indicate that juxtaposition of two GAC core motifs increases their affinity and that the natural element (GACGUGAC) of Dand5 is significantly preferred compared to other GAC-containing RNA motifs.

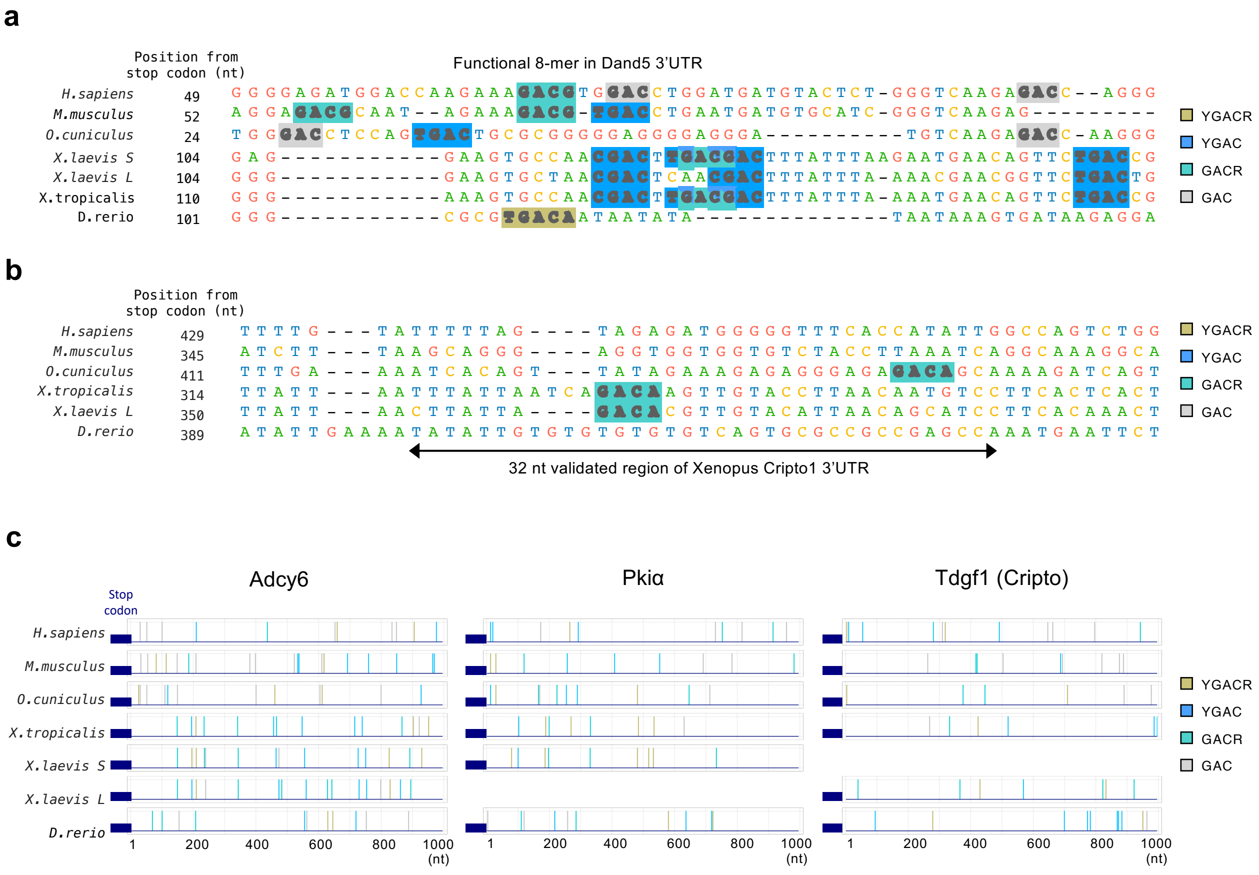

Supplementary Figure 6. Distribution of GAC-containing motifs in the 3'-UTRs of Dand5 and other mRNAs from various vertebrates.

a, b MAFFT-aligned sequences with the inputs of 1000 nt from the stop codon. **a** Mouse *Dand5* and its orthologs. **b** *Cripto* mRNA from various vertebrates. Colored rectangles indicate the Bicc1-binding elements (GACR, YGAC, GAC) identified by RBNS. **c** Distribution of GAC-containing motifs in the 3'-UTR of *Adcy6*, *Pkia,* and *Cripto* mRNAs. Each colored line represents the occurrence of the indicated motif (yellow, YGACR; blue, YGAC; green, GACR; gray, GAC). The GAC motifs are frequently found in the proximal 200-nt region of *Dand5* 3'-UTR across species, but less frequently in *Cripto* 3'-UTRs. A 32-nt region in the *Cripto* 3'-UTR of *Xenopus*, which was previously shown to bind Bicc1, is not highly conserved among vertebrates.

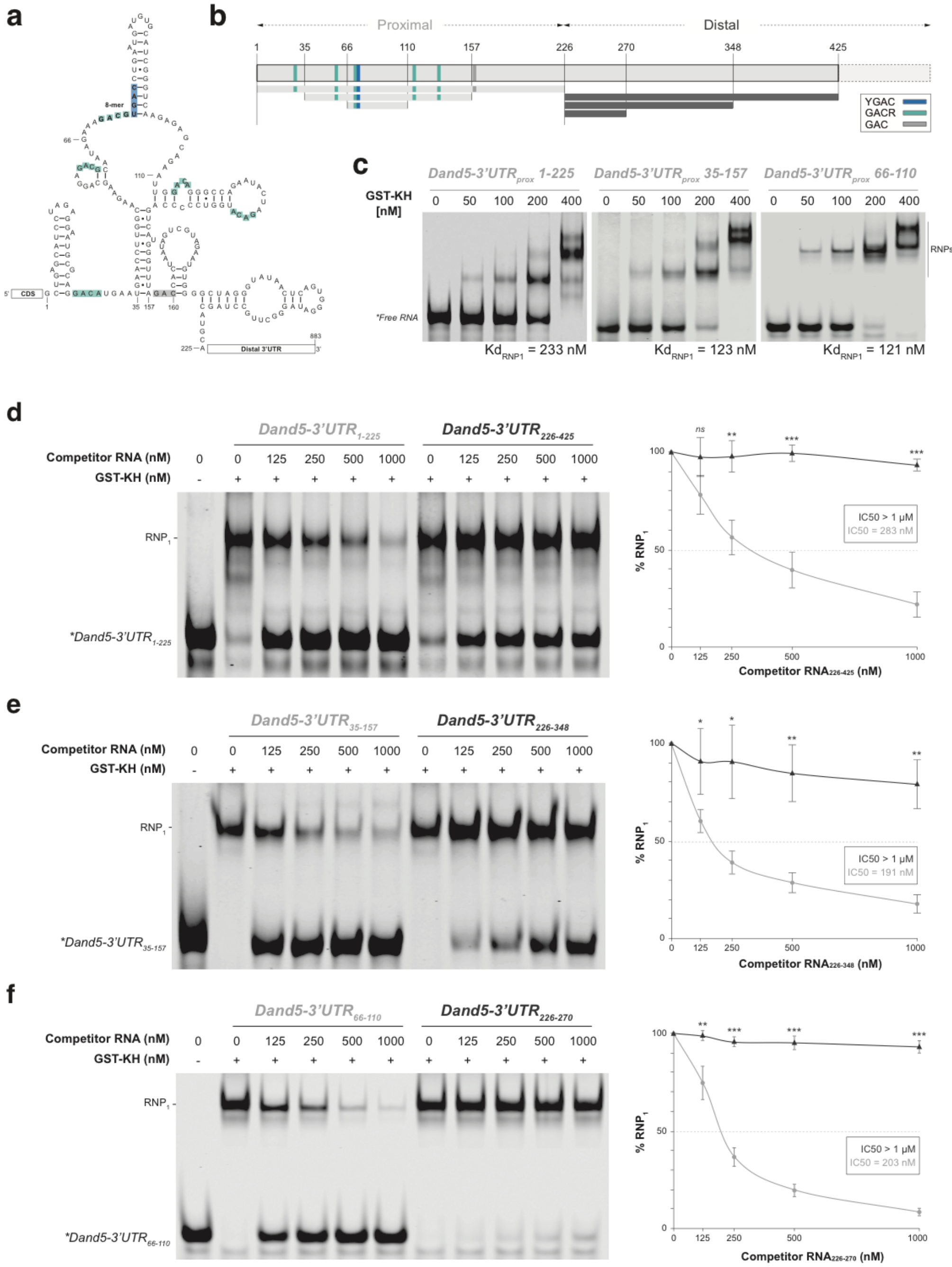

Supplementary Figure 7. Recombinant Bicc1 KH Domains Specifically Bind proximal *Dand5 3'-UTR* Fragments *in vitro*

a Secondary structure predicted for the proximal segment 1-225 of the *Mus musculus Dand5 3'-UTR*. The prediction was performed on http://rna.tbi.univie.ac.at/cgi-bin/RNAWebSuite/RNAalifold.cgi. Coloured boxes highlight short motifs identified by RBNS. **b** Schematic view of the mouse *Dand5 3'-UTR* and proximal and distal fragments with or without GAC motifs. **c** EMSA analysis of increasing amounts of recombinant GST-KH after incubation with labelled RNA probes corresponding to the proximal 3'-UTR fragments. Asterisk denotes unbound probes. Bands of reduced mobility indicate Bicc1 binding. The Kd (given below the gels) was estimated only for the ribonucleoparticle of highest affinity (RNP1). Experiments were done three times with similar results. **d** EMSA analysis of recombinant GST-KH with the indicated fluorescent probes (**Dand5 3'-UTR*) that were pre-assembled before incubation with increasing concentrations of non-fluorescent competitor RNA from the same segment or with similarly sized non-specific distal fragments. The % of RNP_1 was plotted relatively to the competitor RNA concentration. The half maximal inhibitory concentration (IC50) of each competitor RNA is shown in the graphs to the right. Data in (**d**) to (**f**) represent means ± SD from three independent experiments. *$p < 0.05$, **$p < 0.01$, ***$p < 0.001$ (Two-sided Student's t-test). Source data are provided as a Source Data file.

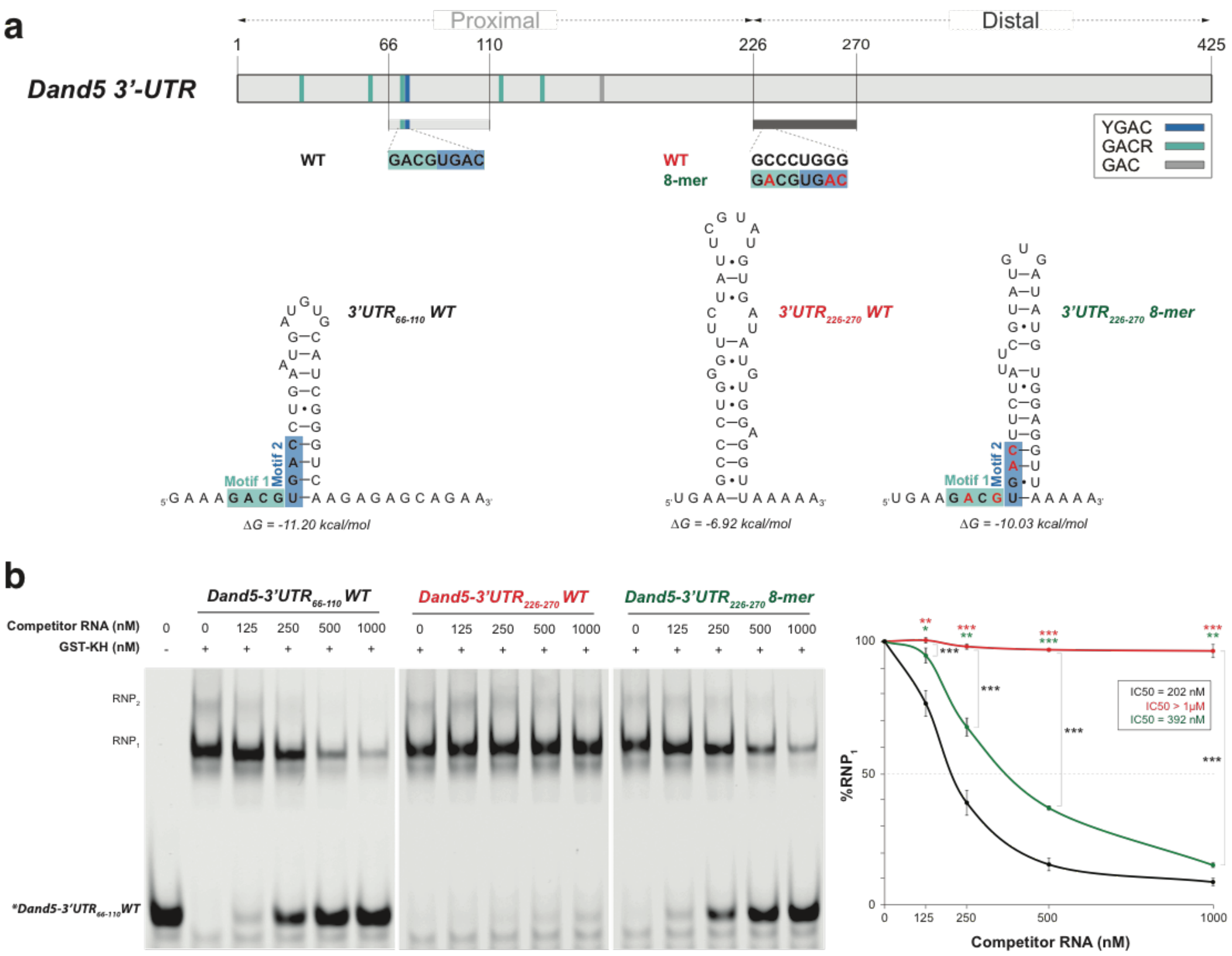

Supplementary Figure 8. Insertion of the proximal GACGUGAC motif after two adenosines confers Bicc1 binding activity to a distal *Dand5 3'-UTR* fragment

a Schematic view of a predicted RNA hairpin at position 227-270 in the distal *Dand5 3'-UTR* fragment that was mutated into a stem-loop containing the bipartite GAC motif AAGACGUGAC from the proximal segment 66-110 to analyze its binding to Bicc1 KH domains in competitive EMSA. Stem-loops in the WT and mutant *Dand5* fragments and their minimum free energies were predicted by the RNAfold server (http://rna.tbi.univie.ac.at/cgi-bin/RNAWebSuite/RNAfold.cgi). **b** Competitive EMSA analysis of the specificity of Bicc1 binding. To monitor competition, the unlabelled *Dand5 3'-UTR*$_{226\text{-}270}$ fragment without (WT) or with the ectopically engineered Bicc1 binding element (*Dand5 3'-UTR*$_{226\text{-}270}$*8-mer*) was titrated on pre-assembled complexes of recombinant GST-KH with the fluorescently labelled proximal *Dand5 3'-UTR*$_{66\text{-}110}$ probe (**Dand5 3'-UTR*$_{66\text{-}110}$). The % of RNP_1 relative to the concentration of the indicated unlabelled competitor RNAs, and the half maximal inhibitory concentrations (IC50) of each competitor are shown to the right. P-values shown above the curves compare each mutant with the WT RNA, whereas comparisons between mutants are indicated by a lateral brace. Data are means ± SD from three independent experiments. *p < 0.05, **p < 0.01, ***p < 0.001 (Two-sided Student's t-test). Source data are provided as a Source Data file.

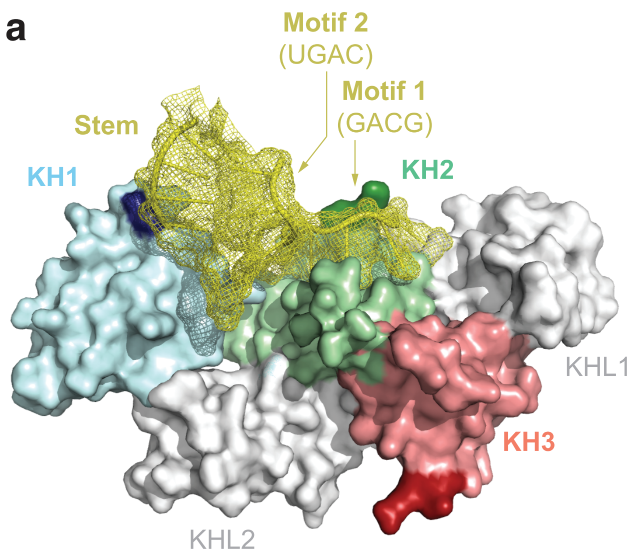

Supplementary Figure 9. Two-step interaction model for the specific recognition of the extended AAGACGUGAC motif by Bicc1 KH1 and KH2 domains

a Model of an RNA hairpin bound at its base to only the KH2 (left) or, after melting of the stem, to KH2 and KH1 domains of Bicc1 (right). The structure of the Bicc1 KH domain repeat has been modelled previously (Rothé et al., 2015). The RNA was docked in the RNA-binding grooves by alignment with the solution structure of the third KH domain of KSRP bound to a G-rich target sequence (PBD: 4B8T) using the PyMol program (Schrödinger). Docking was done manually without further steps of energy minimization.

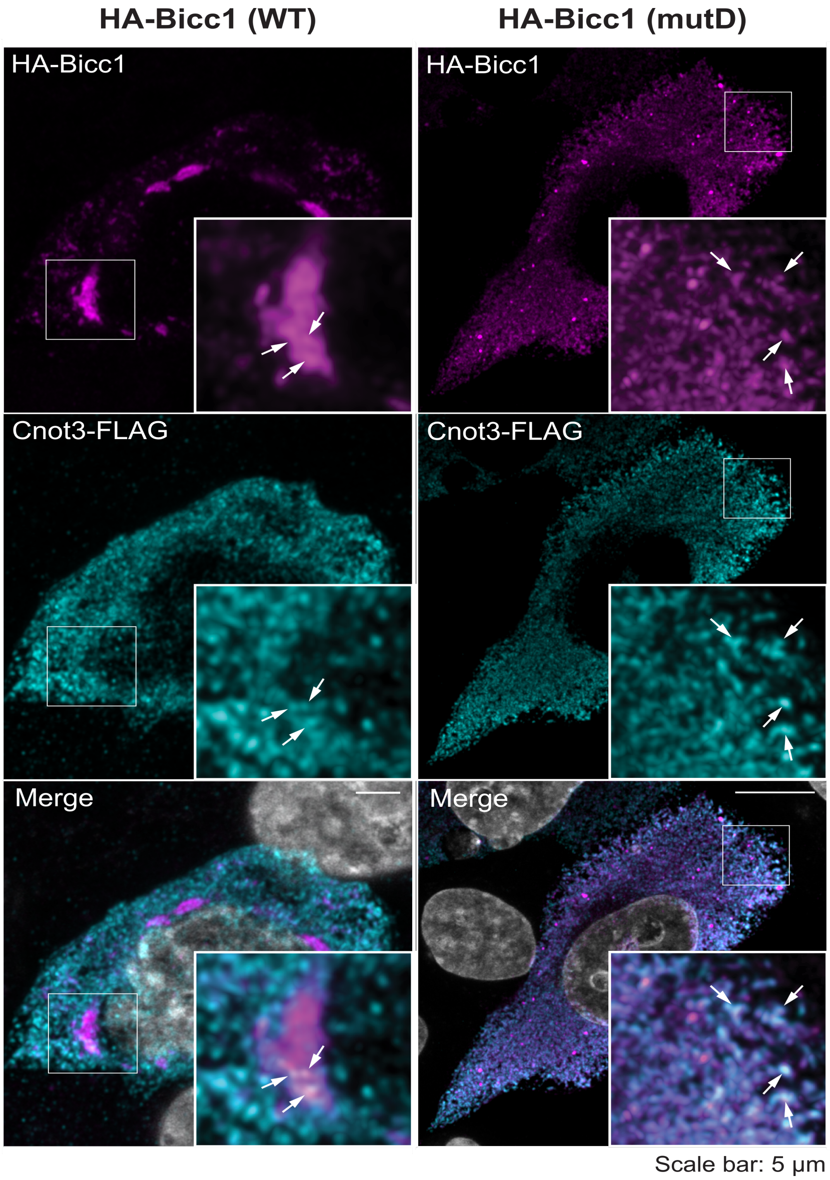

Supplementary Figure 10. Co-localization of exogenously expressed Bicc1 and Cnot-3 proteins in cultured cells.

Immunofluorescence staining of Cnot3-FLAG and HA-Bicc1 (WT or mutD) in transfected HeLa cells. Arrows in magnified areas (insets) indicate overlap of fluorescence signals. Scale bars, 5 μm. Experiments were done at least three times with similar results.

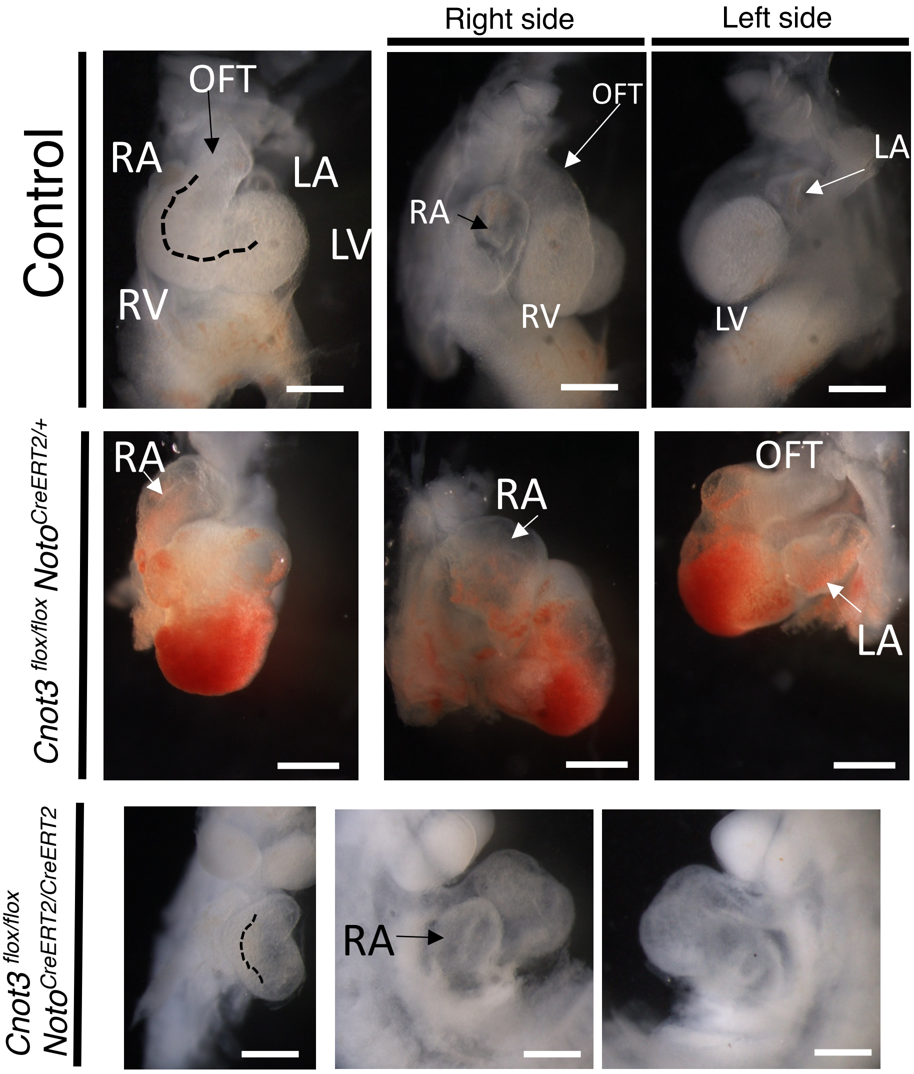

Heart Looping	Control	*Cnot3 CKO*
		Cnot3$^{flox/flox}$ *Noto*$^{CreERT2/+ \text{ or } CreERT2}$
D-Loop (Normal)	25	23
incomplete looping (Abnormal)	0	2
L-Loop (Abnormal)	0	0
Total	25	25

Supplementary Figure 11. Heart looping defects in *Cnot3* CKO mutant mouse embryos. *Cnot3* CKO (*Cnot3*$^{flox/flox}$, *Noto*$^{CreERT2/+ \text{ or } CreERT2/CreERT2}$) or control (*Cnot3*$^{flox/+}$) embryos were treated *in utero* with 5mg tamoxifen 24 and 12 h before the late headfold stage (E7.75), followed by examination of their heart morphologies between E10.5 and E11.5. Images shown are representative of 25 embryos obtained by three independent experiments. In the two representative *Cnot3* CKO embryos shown in the middle and bottom rows, heart looping is abnormal. LA, left atrium; LV, left ventricle; OFT, outflow tract; RA, right atrium; RV, right ventricle. The number of the embryos examined and that of embryos showing abnormal heat looping are summarized below. Scale bars, 500 μm.

Supplementary References

1. Rothe, B. *et al*. Bicc1 Polymerization Regulates the Localization and Silencing of Bound mRNA. *Mol Cell Biol* **35**, 3339-3353 (2015).
