# Supplementary material for: Fluid flow-induced left-right asymmetric decay of Dand5 mRNA in the mouse embryo requires a Bicc1-Ccr4 RNA degradation complex
Source: Nat Commun. 2021 Jul 1;12:4071. doi: 10.1038/s41467-021-24295-2 (PMC8249388; doi:10.1038/s41467-021-24295-2)
Supplement: Supplementary file 2 — Reporting Summary [file 41467_2021_24295_MOESM2_ESM.pdf]

nature research

Corresponding author(s): Hiroshi Hamada, NCOMM-20-01699A

Last updated by author(s): May 6, 2021

Reporting Summary

Nature Research wishes to improve the reproducibility of the work that we publish. This form provides structure for consistency and transparency in reporting. For further information on Nature Research policies, see our Editorial Policies and the Editorial Policy Checklist.

Statistics

For all statistical analyses, confirm that the following items are present in the figure legend, table legend, main text, or Methods section.

n/a	Confirmed	
☐	☒	The exact sample size (*n*) for each experimental group/condition, given as a discrete number and unit of measurement
☒	☐	A statement on whether measurements were taken from distinct samples or whether the same sample was measured repeatedly
☐	☒	The statistical test(s) used AND whether they are one- or two-sided<br>*Only common tests should be described solely by name; describe more complex techniques in the Methods section.*
☒	☐	A description of all covariates tested
☒	☐	A description of any assumptions or corrections, such as tests of normality and adjustment for multiple comparisons
☐	☒	A full description of the statistical parameters including central tendency (e.g. means) or other basic estimates (e.g. regression coefficient) AND variation (e.g. standard deviation) or associated estimates of uncertainty (e.g. confidence intervals)
☐	☒	For null hypothesis testing, the test statistic (e.g. F, t, r) with confidence intervals, effect sizes, degrees of freedom and P value noted<br>*Give P values as exact values whenever suitable.*
☒	☐	For Bayesian analysis, information on the choice of priors and Markov chain Monte Carlo settings
☒	☐	For hierarchical and complex designs, identification of the appropriate level for tests and full reporting of outcomes
☒	☐	Estimates of effect sizes (e.g. Cohen's *d*, Pearson's *r*), indicating how they were calculated

Our web collection on statistics for biologists contains articles on many of the points above.

Software and code

Policy information about availability of computer code

Data collection	FIJI v2.1.0/1.53c,
Data analysis	Cutadapt 1.10<br>Weblogo 3.7.4 [http://weblogo.threeplusone.com/create.cgi]<br>LocARNA 1.9.1 [https://rna.informatik.uni-freiburg.de/LocARNA/Input.jsp],<br>MAFFT v7 [https://www.ebi.ac.uk/Tools/msa/mafft/]<br>MAFFT v7 at the MPI Bioinformatics Toolkit [https://toolkit.tuebingen.mpg.de/tools/mafft]<br>Clustal Omega 1.2.4 [https://www.ebi.ac.uk/Tools/msa/clustalo/]<br>Fiji/Image J Version1.0, MATLAB software, RStudio Version 1.3.1093

For manuscripts utilizing custom algorithms or software that are central to the research but not yet described in published literature, software must be made available to editors and reviewers. We strongly encourage code deposition in a community repository (e.g. GitHub). See the Nature Research guidelines for submitting code & software for further information.

Data

Policy information about availability of data

All manuscripts must include a data availability statement. This statement should provide the following information, where applicable:

- Accession codes, unique identifiers, or web links for publicly available datasets
- A list of figures that have associated raw data
- A description of any restrictions on data availability

Sequencing data of this study were deposited to the Gene Expression Omnibus (GEO) database under accession number GSE140931. The custom script of K-mer

analysis is available in the Github page (https://github.com/KRK13/Kmer2021). Publicly available data was downloaded from NCBI Data Base (https://www.ncbi.nlm.nih.gov) and UCSC Table Browser (https://genome.ucsc.edu). Dand5 sequences were obtained from the NCBI Data Base. Mouse 3'-UTR sequences (GRCm38/mm10) were obtained from the UCSC Table Browser by specifying the track as ALL GENCODE V22 and the table as Basic. Raw data for biochemical (PCR, CoIP) experiments are provided in Supplementary Table 2 and Source Data. Raw data for other experiments are available on request.

Field-specific reporting

Please select the one below that is the best fit for your research. If you are not sure, read the appropriate sections before making your selection.

☒ Life sciences ☐ Behavioural & social sciences ☐ Ecological, evolutionary & environmental sciences

For a reference copy of the document with all sections, see nature.com/documents/nr-reporting-summary-flat.pdf

Life sciences study design

All studies must disclose on these points even when the disclosure is negative.

Sample size	No previous study was used to determine the number of samples. The majority of the experiments were performed in three-four replicates Expression pattern was confirmed with multiple (5 or more in general) embryos at each developmental stages and genotype.
Data exclusions	No data were excluded from the analysis.
Replication	The majority of the experiments were done at least three independent replicates. Gene expression pattern in embryos was confirmed with multiple (5 or more in general) embryos at each developmental stage and genotype.
Randomization	Samples were grouped by their developmental stages and genotype. Otherwise, there was no bias in sample allocation.
Blinding	Researchers were not blinded during experiments and data analysis because proper controls were used. Phenotype of mouse embryo was examined before knowing its genotype, which would make a blind condition.

Reporting for specific materials, systems and methods

We require information from authors about some types of materials, experimental systems and methods used in many studies. Here, indicate whether each material, system or method listed is relevant to your study. If you are not sure if a list item applies to your research, read the appropriate section before selecting a response.

Materials & experimental systems

n/a	Involved in the study
☐	☒ Antibodies
☐	☒ Eukaryotic cell lines
☒	☐ Palaeontology and archaeology
☐	☒ Animals and other organisms
☒	☐ Human research participants
☒	☐ Clinical data
☒	☐ Dual use research of concern

Methods

n/a	Involved in the study
☒	☐ ChIP-seq
☒	☐ Flow cytometry
☒	☐ MRI-based neuroimaging

Antibodies

Antibodies used	1) Anti-Bicc1 (HPA045212, Sigma-Aldrich), 1:100<br>2) Anti-acetylated tubulin (T6793, Sigma-Aldrich), 1:100<br>3) Anti-ZO-1 (33-9100, clone ZO1-1A12, Invitrogen), 1:10<br>4) Anti-Odf2 (ab43840, Abcam), 1:100<br>5) Anti-HA beads (A2095, Sigma-Aldorich). The detailed conditions used for protein co-immunoprecipitation and RNA co-immunoprecipitation are described in Methods.<br>6) Anti-Cnot3 (generated by T.Y and described in Suzuki et al Development, 2019), 1:50-100<br>7) Anti-FLAG (Sigma, #F1804). The detailed conditions used for RNA co-immunoprecipitation are described in Methods.
Validation	Specificity of antibodies has been validated by manufacturers. 1)https://www.sigmaaldrich.com/catalog/product/sigma/hpa045212?lang=en&region=CA , 2) https://www.sigmaaldrich.com/catalog/product/sigma/t6793?lang=en&region=US, 3)https://www.thermofisher.com/antibody/product/ZO-1-Antibody-clone-ZO1-1A12-Monoclonal/33-9100 , 4)https://www.abcam.co.jp/cenexin1odf2-antibody-ab43840.html, 5) https://www.sigmaaldrich.com/catalog/product/sigma/a2095?lang=en&region=CA.<br><br>Specificity of anti-Bicc1 antibody and anti-Cnot3 antibody was validated with Bicc1(-/-) and Cnot3 (-/-) embryos and mice by the authors.

Eukaryotic cell lines

Policy information about cell lines

Cell line source(s)	HEK293T and HeLa cells were obtained from ATTC (https://www.atcc.org/products/all/crl-3216.aspx, https://www.atcc.org/products/all/CCL-2.aspx).
Authentication	These cell lines have been authenticated by ATTC (https://www.atcc.org/products/all/crl-3216.aspx, https://www.atcc.org/products/all/CCL-2.aspx).
Mycoplasma contamination	Negative for mycoplasma contamination
Commonly misidentified lines (See ICLAC register)	None

Animals and other organisms

Policy information about studies involving animals; ARRIVE guidelines recommended for reporting animal research

Laboratory animals	Genetically engineered mice used in this study has C57Bl/6J background. Mice were maintained under 12/12 hour light/dark cycle, humidity between 40-60 % , temperature 20-25C. Embryos were obtained at the indicated stage (usually 8 days after fertilization) upon natural mating between males and females.
Wild animals	Wild animals were not used in this study.
Field-collected samples	No field-collected samples were used.
Ethics oversight	All animal experiments were approved by the Institutional Animal Care and Use Committees (IACUCs) of RIKEN Kobe Branch.

Note that full information on the approval of the study protocol must also be provided in the manuscript.
